# Mechanically adaptive Mg-Ti composites guided by single-cell insights accelerate load-bearing bone regeneration via dual modulation of osteogenesis and osteoclastogenesis

**DOI:** 10.1016/j.bioactmat.2025.10.038

**Published:** 2025-11-06

**Authors:** Wanxin Zheng, Sirui Tian, Jiaxing Huo, Qiyue Zhang, Danning Wang, Zengqian Liu, Baohong Zhao, Yuzhong Gao, Zhefeng Zhang, Qiang Wang

**Affiliations:** a Liaoning Provincial Key Laboratory of Oral Diseases, China Medical University, Shenyang, Liaoning, 110001, China; b Department of Oral Implantology, School and Hospital of Stomatology, China Medical University, Shenyang, Liaoning, 110001, China; c Institute of Metal Research, Chinese Academy of Sciences, Shenyang, Liaoning, 110016, China; d The First Affiliated Hospital of Jinzhou Medical University, Jinzhou, Liaoning, 121001, China

**Keywords:** Single-cell sequencing, Mg-Ti composites, Osteoclast regulation, Bone regeneration, PDGF-BB

## Abstract

The regeneration and repair of load-bearing bone defects require a delicate balance between mechanical stability and biological integration. Magnesium (Mg)-based materials are promising due to their bioactivity, but their rapid degradation often results in the premature loss of mechanical strength, which compromises their ability to provide structural support during the critical early stages of bone healing, particularly in load-bearing applications. To address this challenge, a Magnesium-Titanium (Mg-Ti) composite was designed, integrating the bioactivity of Mg with the mechanical stability of titanium (Ti). Single-cell RNA sequencing revealed that Mg selectively promoted Mesenchymal Stem Cells (MSCs) recruitment and osteogenic differentiation, while also arresting osteoclast precursors to retain a less differentiated state. These precursors secreted PDGF-BB, coupling osteogenesis with angiogenesis. The Ti scaffold, fabricated as a 3D-printed rhombic dodecahedron structure that mimicked trabecular bone, ensured mechanical support while allowing controlled Mg degradation. This design enabled the progressive adaptation of the composites' mechanical properties to those of natural bone over time, in accordance with Nielsen's law, thereby optimizing both short-term stability and long-term integration. Mg-Ti-1200 exhibited dual-regulatory effects of osteogenesis-osteoclastogenesis: enhancing MSCs osteogenesis via the PI3K-Akt pathway, while inhibiting osteoclast maturation through the PLCγ2-Calcineurin-NFATc1 pathway. *In vivo*, the Mg-Ti-1200 resulted in a 55 % increase in bone volume and exhibited mechanical properties comparable to those of natural bone after 8 weeks of implantation. This study presented a mechanism-guided biomaterial strategy that integrated both mechanical and biological optimization for the functional regeneration of load-bearing bone defects.

## Highlights

• scRNA-seq revealed Mg selectively promotes MSCs recruitment and osteogenesis while inhibiting osteoclastogenesis.
• Mg-Ti-1200 composite arrests osteoclasts' differentiation , promoting bone-vascular coupling through PDGF-BB.
• Mg-Ti-1200 composite enables controlled degradation with gradual elastic modulus matching.

## Graphical abstract

**Figure:**
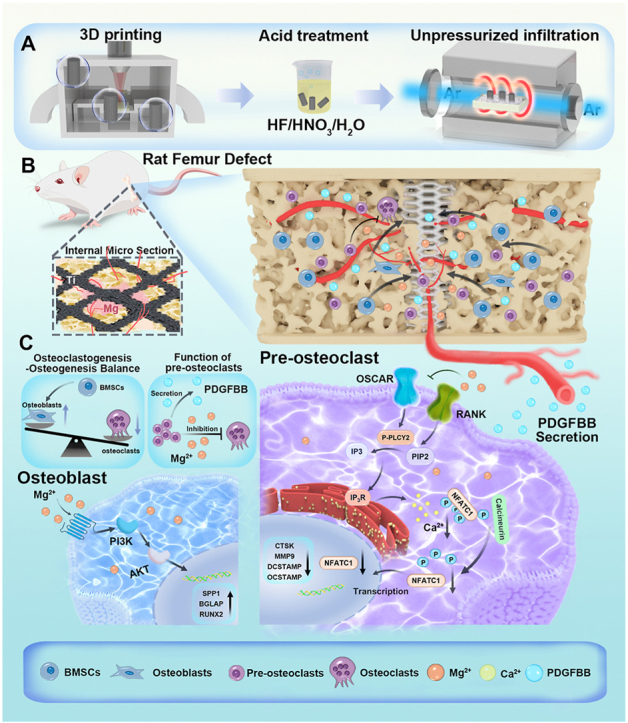

## 1 Introduction

The effective repair of load-bearing bone defects remains a critical challenge in orthopedic practice [[1], [2], [3]]. Particularly in scenarios requiring extensive bone replacement and structural reconstruction, achieving a coordinated balance between mechanical stability and biological integration represents a central challenge shared by both material design and regenerative strategies [4,5]. Although conventional metallic materials such as Ti alloys exhibit excellent mechanical properties capable of sustaining long-term load-bearing demands, their inherent bioinertness and non-degradability limit deep integration with host tissue [[6], [7], [8], [9]]. This often leads to adverse outcomes such as stress shielding, interfacial osteolysis, and even early implant loosening and failure-complications that are especially pronounced in complex mechanical environments, highlighting the fundamental limitations of traditional bone repair approaches [10,11].

Recent advances in bone tissue engineering and regenerative medicine have shifted the paradigm of bone regeneration [12,13]. It is now well recognized that successful bone healing depends not only on the activation of osteogenic cells but also on the finely tuned interplay among osteogenesis, osteoclastogenesis, and angiogenesis [[14], [15], [16]]. Most current biomaterial strategies primarily focus on promoting osteoblastic activities-such as cell adhesion, proliferation, and differentiation, while largely neglecting the dynamic nature of bone regeneration. In this context, the osteoclast lineage plays a pivotal regulatory role [[17], [18], [19]]. Traditionally regarded as mere “destructive” cells, recent studies have revealed that osteoclast precursors at specific differentiation stages exhibit potent paracrine functions [20]. Notably, they secrete coupling factors such as Platelet-Derived Growth Factor-BB (PDGF-BB), which promote angiogenesis and recruit osteoprogenitor cells, thus serving as critical modulators at the bone-vascular interface [[20], [21], [22]]. Therefore, instead of broadly inhibiting osteoclast activity, a more refined approach that targets the lineage-specific fate of osteoclast precursors, particularly preserving the pre-osteoclast state has emerged as a promising strategy to construct an optimal microenvironment for bone regeneration [20,23].

Within this framework, Mg and its degradation product Mg^2+^ have garnered significant attention as emerging bioactive agents [[24], [25], [26]]. Mg not only exhibits intrinsic osteoinductive properties but also exerts dose-dependent regulatory effects on osteoclasts differentiation [26,27]. At appropriate concentrations, Mg can suppress terminal osteoclast maturation while preserving precursor phenotypes and activating multiple signaling pathways related to osteogenesis and angiogenesis [27,28]. These findings suggest that Mg may promote bone-vascular coupling through a mechanism of reversible osteoclast inhibition. However, the rapid *in vivo* degradation of metallic Mg presents challenges in maintaining localized and controlled Mg^2+^ release, potentially leading to adverse effects such as local alkalization and gas accumulation [29,30]. These issues underscore the need for biomaterials capable of achieving both mechanical support and precise biological modulation. Developing functionalized composite with spatially and temporally controllable Mg^2+^ release thus represents a critical direction for next-generation systems aimed at load-bearing bone repair.

To investigate Mg's lineage-specific effects during early bone healing while circumventing confounding factors from the initial burst release, we implanted pure Mg rods into a murine femoral defect model and conducted single-cell RNA sequencing (scRNA-seq) on day 7 [[31], [32], [33], [34]], revealing that Mg orchestrated dual modulation of osteogenesis-osteoclastogenesis via MSCs recruitment and osteoclastogenic arrest, manifested as reduced mature osteoclasts and expanded osteoclast precursors, perpetuating paracrine precursors and initiating bone-vascular cascades. Capitalizing on these insights, we engineered a synergistic composite: 3D-printed Ti scaffolds featuring rhombic dodecahedral architectures with degradable Mg phases, optimized via three distinct pore sizes (900, 1200, 1500 μm) for Mg degradation and mechanical adaptability, enabling sustained Mg^2+^ release to modulate osteoclast-osteoblast-endothelial crosstalk. The rhombic dodecahedral geometry was strategically selected due to its space-filling capacity, isotropic mechanical properties, and stress distribution characteristics that mimicked trabecular bone while minimizing stress concentration under physiological loading. Acid etching enhanced Ti scaffolds' surface roughness for osteoblast adhesion, while degradation byproducts propelled deep migration and bone ingrowth. This Mg-driven modulation arrested osteoclasts, curtailing resorption yet upregulating PDGF-BB to synergize with endothelial cells for neovascular-osteoblast migration, deep integration, and nutrient remodeling, culminating in Mg-Ti interface evolution to triphasic (Mg-Ti-Bone) then Ti-Bone biphasic with biomimetic mechanics, alleviated stress shielding, and enhanced load-bearing restoration. Through multiscale cellular-tissular-material orchestration, this study elucidates an innovative strategy that modulates of osteogenesis-osteoclastogenesis and activates bone-vascular coupling to enable mechanically adaptive repair of load-bearing bone defects.

## 2 Results and discussion

### 2.1 Single-cell RNA sequencing

**Fig. 1:**
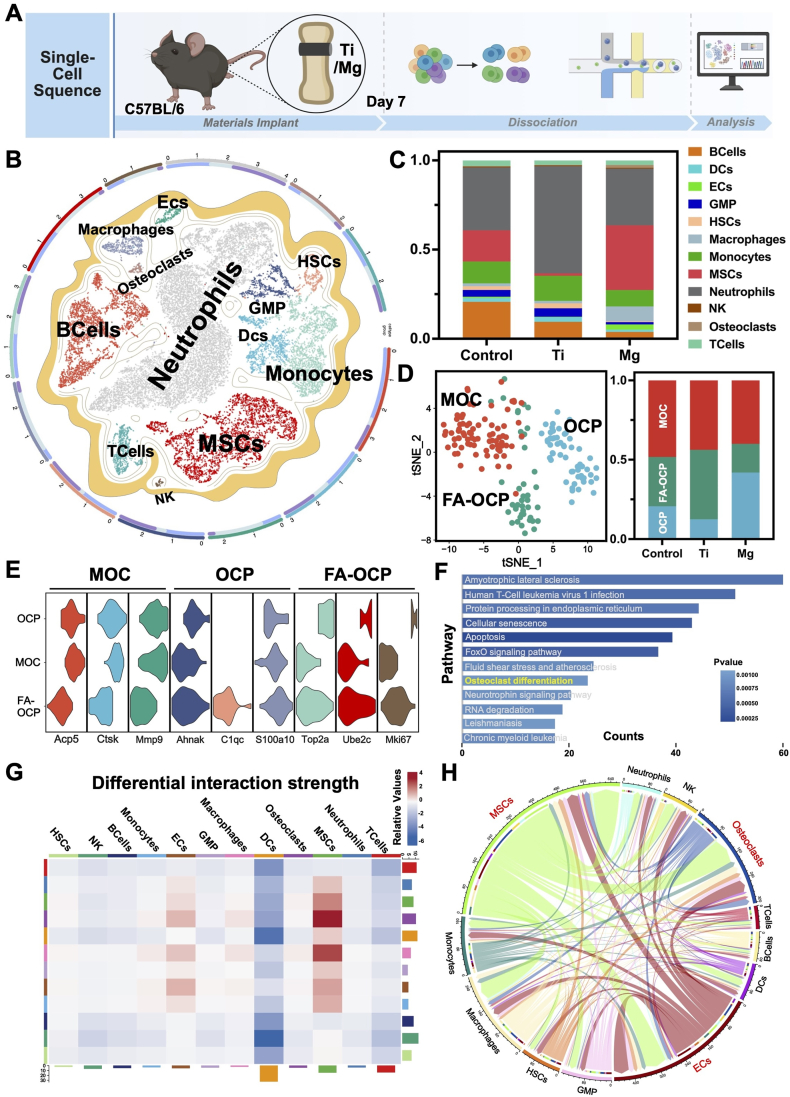
**Single-cell RNA sequencing profiling of peri-implant tissues 7 days post-implantation of Control, pure Ti, or pure Mg implants in C57BL/6 mice:** (A) Schematic of experimental workflow: implantation, tissue harvesting, and scRNA-seq pipeline. (B) t-SNE projection of major cell populations: neutrophils, macrophages, osteoclasts, monocytes, DCs, GMPs, NKs, HSCs, MSCs, B cells, T cells, and ECs. (C) Bar graph showing proportional distribution of cell types across groups. (D) UMAP of osteoclast subpopulations, with bar graphs quantifying OCPs, MOCs, and FA-OCPs proportions. (E) Violin plots of marker gene expression across osteoclast clusters. (F) KEGG enrichment of DEGs in osteoclasts (Mg vs. Control). (G) Heatmap of intercellular interaction strengths in Mg group vs. Control. (H) Circular diagram of altered communication networks in Mg-implanted microenvironment vs. Control.

To investigate how metallic implants influenced early-stage bone microenvironments and lineage-specific cellular responses, we performed scRNA-seq on peri-implant femoral tissues from C57BL/6 mice treated with pure Ti, pure Mg, or left untreated (Control) (Fig. 1A). t-Distributed Stochastic Neighbor Embedding (t-SNE) dimensionality reduction revealed twelve distinct cellular clusters across the three groups, encompassing MSCs, Endothelial Cells (ECs), osteoclast-lineage cells, and various stromal populations (Fig. 1B, Fig. S1). The Mg group showed a marked increase in MSCs, indicating an enhanced recruitment of osteogenic progenitors (Fig. 1C). Gene expression profiling across groups further demonstrated that osteogenic markers *Runx2* and *Bglap* were most highly expressed in the Mg group, with *Runx2* levels in Ti notably higher than in Control, while the fibrotic marker *α-SMA* was significantly upregulated in Control, indicating a fibrotic bias (Fig. S2A). Subpopulation clustering further revealed MSCs heterogeneity, with Control exhibiting a higher profibrotic MSCs (pFBs) fraction and reduced osteoblast/pre-osteoblast proportions compared to Ti, whereas Mg displayed the lowest profibrotic and highest osteogenic fractions (Fig. S2B–D). Additionally, Subclustering of osteoclast-lineage cells identified three developmental states: Osteoclast Precursors (OCPs), Fusion-Activated OCPs (FA-OCPs), and Mature Osteoclasts (MOCs) [35] (Fig. 1D, E, Fig. S3). Mg implants resulted in a significant expansion of the OCPs population and a corresponding reduction in MOCs, suggesting that Mg might inhibit osteoclast maturation at the early stage of bone healing. Kyoto Encyclopedia of Genes and Genomes (KEGG) analysis confirmed a significant downregulation of the osteoclast differentiation pathway in the Mg group compared to Control, whereas Ti had minimal impact (Fig. 1F, Fig. S4).

Notably, enhanced ligand-receptor interactions were observed between osteoclast and both MSCs and ECs in the Mg group, pointing to an active coupling between osteoclast and osteogenic or angiogenic cell populations (Fig. 1G and H). Rather than progressing toward terminal differentiation, osteoclast under Mg exposure might serve as regulatory intermediates that coordinated bone formation and neovascularization. These findings suggested that Mg implants promoted a favorable shift in the modulation of osteogenesis and osteoclastogenesis, facilitating early stage osteogenic activation through precursor-mediated intercellular crosstalk.

### 2.2 Fabrication and characterization of Mg-Ti composites

**Fig. 2:**
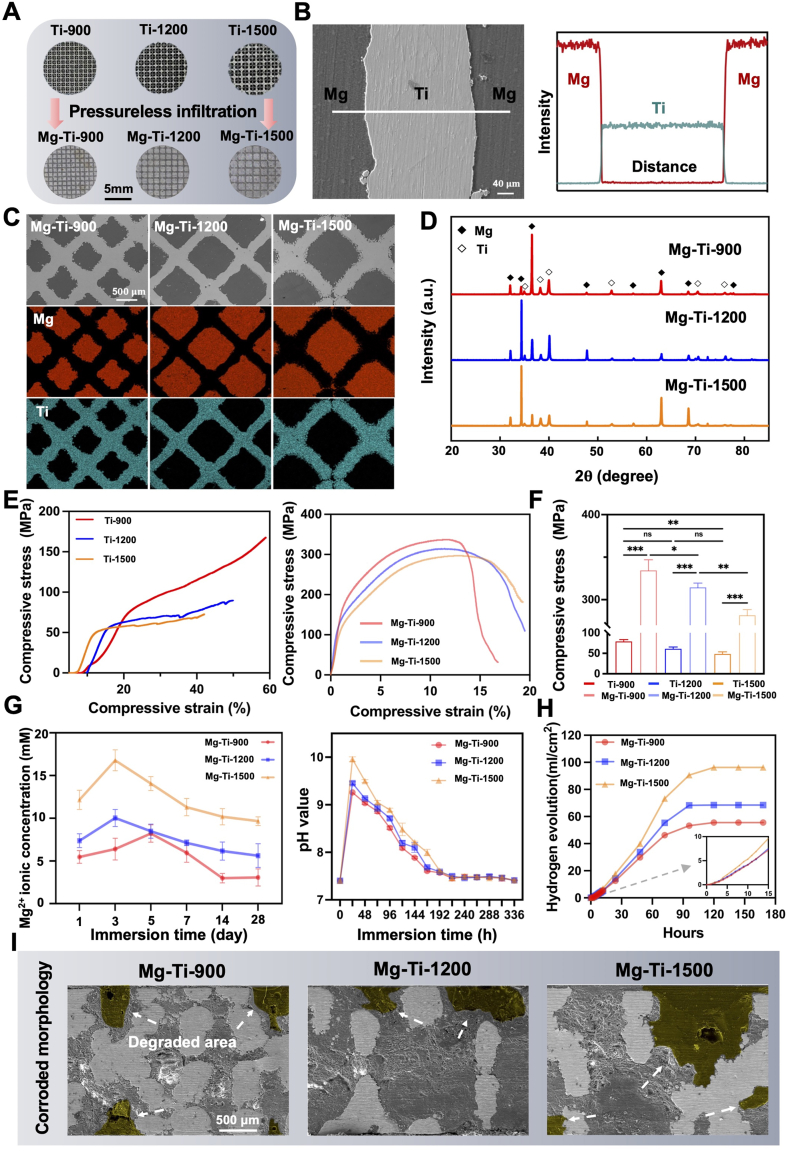
**Microstructure and corrosion behavior of Mg-infiltrated porous Ti scaffolds with different architectures:** (A) Schematic of porous Ti scaffolds and corresponding Mg-infiltrated composites (Scale bars: 5 mm). (B) SEM line scan across the Mg/Ti interface (Scale bars: 40 μm). (C) Cross-sectional SEM images and EDS elemental maps of Mg-Ti composites (Scale bars: 500 μm). (D) XRD patterns of different Mg-Ti composites. (E) Compressive stress-strain curves of Ti scaffolds and Mg-Ti composites. (F) Compressive strength statistics for Ti scaffolds and Mg-Ti composites. The data were shown as the mean ± SD (*n* = 6); ∗*P* < 0.05; ∗∗*P* < 0.01; ∗∗∗*P* < 0.001 indicated significant differences between the indicated columns (one-way ANOVA). (G) Mg^2+^ release and pH evolution of different Mg-Ti composites in simulated body fluid (SBF). (H) Hydrogen evolution profiles of Mg-Ti composites in SBF; inset shows initial release kinetics. (I) SEM images of Mg-Ti composites after 7 days in SBF, yellow pseudo-color indicates fully degraded areas. (Scale bars: 500 μm).

To construct a biomaterial platform that can promote bone regeneration and regulate the osteogenesis and osteoclastogenesis, while meeting the dual demands of mechanical performance and controlled degradation behavior in load bearing bone defect repair, this study employed the Pressureless Infiltration method to melt and infiltrate pure Mg in a proper temperature into pure Ti scaffolds featuring rhombic dodecahedral architectures with three different pore sizes (porous 900 **μm**, porous 1200 **μm**, porous 1500 **μm**) [36,37] (Figs. S5 and S6, Table S1). This resulted in the successful fabrication of Mg-Ti composites with a complete structure and well-bonded interfaces (Fig. 2A and B, Fig. S7). The composites corresponding to the three pore sizes were named Mg-Ti-900, Mg-Ti-1200, and Mg-Ti-1500. The overall formation was uniform with good geometric consistency.

Scanning Electron Microscopy (SEM) and Energy-Dispersive X-ray Spectroscopy (EDS) line scan analyses revealed that the Mg/Ti interface was smooth and continuous, with no significant diffusion of elements or formation of intermetallic compounds, indicating good interface compatibility (Fig. 2B and C). X-ray Diffraction (XRD) further confirmed that only Mg and Ti diffraction peaks were present in all composites (Fig. 2D), with no detectable Mg-Ti intermetallic phases. In comparison with pure Ti scaffolds, Mg-Ti composites demonstrated a marked enhancement in compressive strength and hydrophilicity (Fig. 2E, F, Fig. S8 and S9). This mechanical performance met the requirements for bearing loads in bone defect regions (Table S2). However, as the Mg phase degrades, the materials' mechanical properties were inevitably altered. Therefore, understanding the specific degradation behavior of each material became particularly important. In our previous work, we employed Nielsen's law to predict and model the elastic modulus evolution during the degradation process of Mg-Ti composites [36]. The results demonstrated that as the Mg phase underwent progressive degradation and facilitated bone ingrowth, the elastic modulus of the Mg-Ti-Bone biocomposite gradually converged toward that of natural bone, ultimately achieving mechanical properties roughly comparable to native bone tissue (Fig. S10). Subsequently, we proceeded to investigate the degradation rates of the materials. Fig. 2G showed the concentration trends of Mg ions after immersion in simulated body fluid for different durations. The Mg ions release concentration of the Mg-Ti-900 and Mg-Ti-1200 groups remained below 10 mM, indicating that their degradation rates were controllable and within the physiological concentration threshold for osteogenesis as reported in the literature [38]. In contrast, the Mg-Ti-1500 group experienced a rapid increase above 10 mM within the first 7 days, maintaining a high plateau, suggesting excessive degradation in the high-porosity structure, which could lead to potential cytotoxicity [24,38].

Additionally, the rapid release of Mg ions was accompanied by an increase in solution pH (Fig. 2G). All groups showed some degree of pH increase, with the Mg-Ti-1500 group exhibiting the most pronounced alkalization effect. The pH significantly increased within 7 days and exceeded the physiological stable range. Fig. 2H illustrated the hydrogen evolution data over 180 h for the three groups. The Mg-Ti-1500 group exhibited the highest hydrogen evolution and a steep increase in the curve, validating that the degradation reaction in the high-porosity structure was more intense, consistent with the trend of Mg ions release. The Mg-Ti-900 and Mg-Ti-1200 groups showed relatively slower hydrogen evolution rates, tending toward a plateau, demonstrating better control over degradation. SEM morphological analysis (Fig. 2I) further corroborated these distinctive degradation characteristics after 7 days' degradation: the Mg-Ti-900 surface maintained relatively intact microstructural features with only sporadic corrosion initiation sites; the Mg-Ti-1200 surface exhibited uniformly distributed localized corrosion regions while overall structural integrity was preserved; whereas the Mg-Ti-1500 surface displayed extensive pitting corrosion and severe structural deterioration, with dramatic microstructural degradation that was highly consistent with the macroscopic degradation behavior. Moreover, corrosion fatigue tests (10 Hz, R = 0.1, 10^6^ cycles) conducted in flowing Ringer's solution revealed no interfacial debonding in Mg-Ti composites, with cross-sectional SEM analysis confirming that the good wettability of Mg on Ti and the continuous interpenetrating structure maintained robust interface integrity despite normal corrosion behavior (Fig. S11).

Collectively, these results demonstrated that pore size exerted a significant regulatory effect on the degradation behavior of Mg-Ti composites. In the relatively confined porous environment of Mg-Ti-900, mass transfer limitations impeded Mg^2+^ migration, establishing a local concentration gradient barrier that subsequently suppressed the sustained progression of electrochemical dissolution. Conversely, in the excessively open structure of Mg-Ti-1500, the elimination of mass transfer constraints resulted in degradation kinetics that were entirely governed by electrochemical reactions, leading to disordered and rapid dissolution that compromised both controllability and predictability. In contrast, the intermediate pore size of Mg-Ti-1200 achieved an optimal balance between mass transfer processes and electrochemical kinetics, maintaining both the stability of ion release and the gradual evolution of structural integrity. This configuration thereby provided a degradation response pattern that was optimally aligned with the temporal functional requirements of bone repair processes.

### 2.3 Biocompatibility and osteogenic effects

**Fig. 3:**
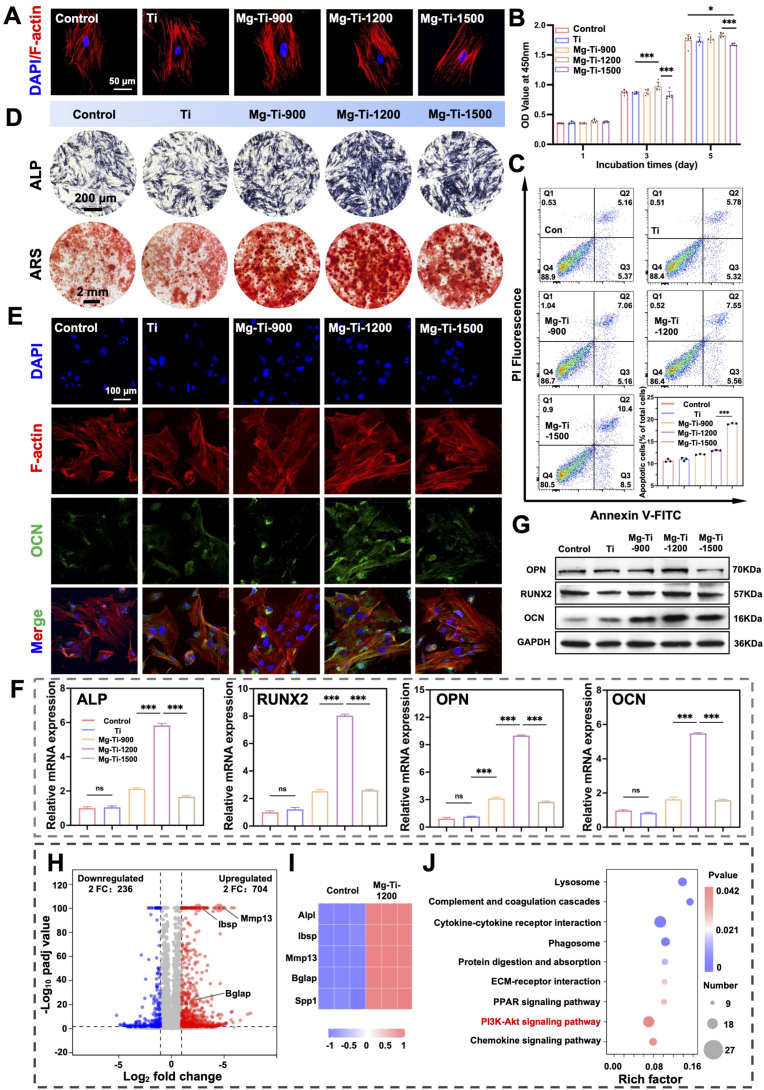
**Osteogenic differentiation of cells cultured on Mg-Ti composites:** (A) Phalloidin staining showing F-actin distribution in the cells (Scale bars: 50 μm). (B) CCK-8 assay to evaluate cell proliferation. The data were shown as the mean ± SD (*n* = 6); ∗*P* < 0.05; ∗∗*P* < 0.01; ∗∗∗*P* < 0.001 indicated significant differences between the indicated columns (one-way ANOVA). (C) ALP (Scale bars: 200 μm) and ARS (Scale bars: 2 mm) staining of BMSCs. (D) Apoptosis analysis via flow cytometry of BMSCs with quantification. The data were shown as the mean ± SD (*n* = 3); ∗*P* < 0.05; ∗∗*P* < 0.01; ∗∗∗*P* < 0.001 indicated significant differences between the indicated columns (one-way ANOVA). (E) Immunofluorescence staining for osteocalcin (OCN) expression (Scale bars: 100 μm). (F) mRNA expression analysis of osteogenic markers (*Alp*, *Runx2*, *Opn*, *Ocn*). The data were shown as the mean ± SD (*n* = 6); ∗*P* < 0.05; ∗∗*P* < 0.01; ∗∗∗*P* < 0.001 indicated significant differences between the indicated columns (one-way ANOVA). (G) Western blot analysis of osteogenic markers (RUNX2, OPN, OCN). (H) Volcano plot in transcriptomics sequencing highlighting upregulated (red) and downregulated (blue) genes between Mg-Ti-1200 and Control. (I) Heatmap showing differentially expressed genes between Mg-Ti-1200 and Control. (J) KEGG pathway enrichment analysis.

Mouse Bone Marrow Stromal Cells (BMSCs) were isolated and characterized for purity and multipotent differentiation potential using established assays (Fig. S12A and B). These validated BMSCs were then employed to assess the bioactivity of Mg-Ti composites. Specifically, to delineate the structure-activity relationships among pore size, Mg ions release, and cellular responses, we systematically investigated the effects of extracts from three Mg-Ti composites with varying pore sizes on BMSCs. Phalloidin staining revealed that Mg-Ti-900 and Mg-Ti-1200 showed no significant differences in cell morphology compared to Control and pure Ti groups, whereas Mg-Ti-1500 exhibited restricted cell spreading and cellular contraction (Fig. 3A), indicating differential cellular microenvironments generated by varying pore sizes. CCK-8 proliferation analysis confirmed the non-linear relationship between Mg ions concentration and cellular responses. Mg-Ti-900 demonstrated a mild promoting effect relative to Control, Mg-Ti-1200 exhibited sustained and significant proliferative advantages, while Mg-Ti-1500 suppressed cell proliferation (Fig. 3B). Flow cytometric apoptosis detection further validated this relationship: Mg-Ti-1500 showed significantly increased early and late apoptotic cells, while Mg-Ti-900 and Mg-Ti-1200 maintained low apoptosis levels (Fig. 3C), confirming that Mg-Ti-1500 pore size exceeded the safe release threshold.

Based on biocompatibility assessment results, we further examined the pore size-dependent efficiency of osteogenic differentiation. Alkaline Phosphatase (ALP) staining revealed a clear dose-response relationship in early osteogenic marker expression: Mg-Ti-1200 > Mg-Ti-900 > Mg-Ti-1500 (Fig. 3D). This non-monotonic relationship was further confirmed by Alizarin Red mineralization staining (ARS), where Mg-Ti-1200 achieved maximum area and most uniform calcium deposition, while Mg-Ti-900 and Mg-Ti-1500 showed limited mineralization (Fig. 3D). These results indicate an optimal Mg ions concentration range where osteogenic effects reach peak levels. Molecular-level analysis revealed the transcriptional regulatory basis of this phenomenon. Immunofluorescence analysis revealed that Mg-Ti-1200 not only exhibited the highest OCN protein expression intensity but also the most organized intracellular distribution pattern (Fig. 3E), indicating that the appropriate Mg ions concentration in Mg-Ti-1200 significantly promoted BMSCs differentiation toward mature osteoblasts. Quantitative Reverse Transcription Polymerase Chain Reaction (qRT-PCR) detection of four key osteogenic genes (*Alp, Runx2, Opn, Ocn*) showed that Mg-Ti-1200 achieved the highest expression levels in all tested genes, significantly exceeding Mg-Ti-900 and Mg-Ti-1500 (Fig. 3F). Western blot protein validation results were consistent with gene expression trends, with Mg-Ti-1200 showing the strongest expression of OPN, RUNX2, and OCN protein levels (Fig. 3G).

To elucidate the molecular mechanisms underlying Mg-Ti-1200 superiority, we performed transcriptomic analysis. Differential gene expression analysis compared to Control identified 704 upregulated and 236 downregulated genes, with osteogenesis-related genes *Alpl*, *Ibsp, Mmp13*, *Bglap* and *Spp1* showing coordinated upregulation patterns (Fig. 3H and I). KEGG pathway enrichment analysis revealed significant activation of the PI3K-Akt signaling pathway (Fig. 3J, Fig. S12C), a key regulatory node for cell survival, proliferation, and differentiation, which has been documented to participate in Mg ion-promoted osteogenic differentiation of BMSCs [14,24,27].

### 2.4 Differential regulation of osteoclast differentiation

**Fig. 4:**
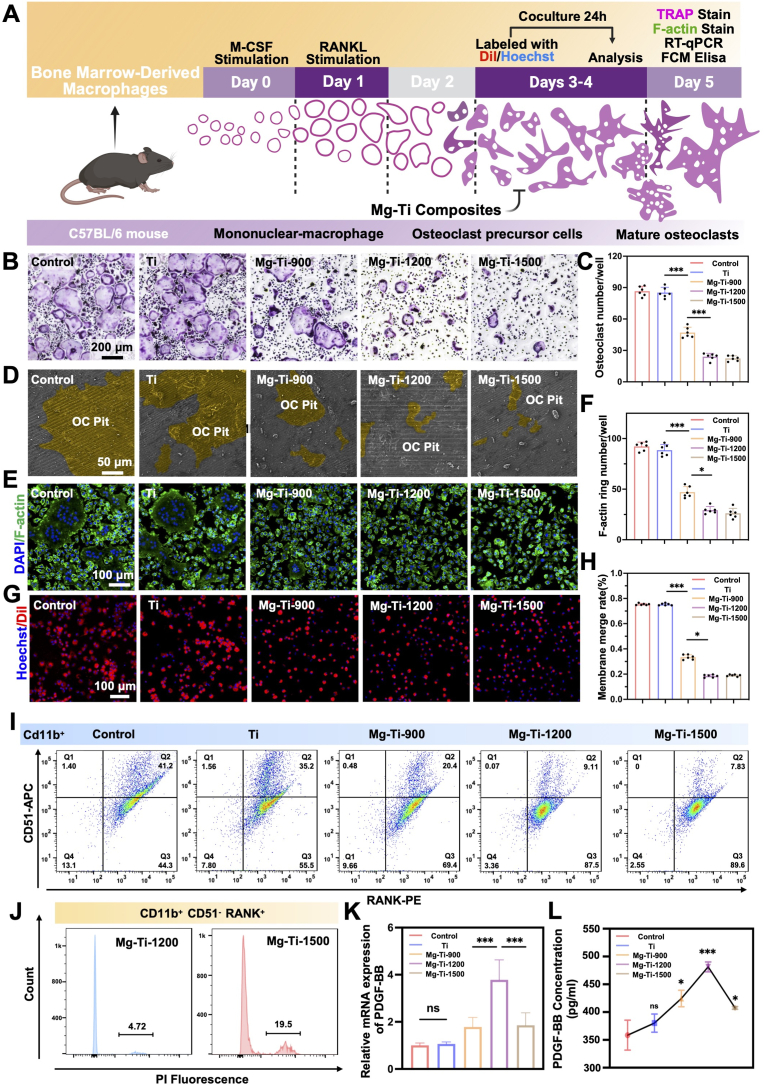
**Evaluation of osteoclastogenesis and bone resorption upon treatment with Mg-Ti composites: (A)** Schematic illustration of osteoclast differentiation from monocytes under M-CSF and RANKL stimulation, with different treatments. **(B)** Representative TRAP staining images of osteoclasts cultured with different groups (Scale bars: 200 μm). **(C)** Quantification of TRAP-positive multinucleated cells. The data were shown as the mean ± SD (*n* = 6); ∗*P* < 0.05; ∗∗*P* < 0.01; ∗∗∗*P* < 0.001 indicated significant differences between the indicated columns (one-way ANOVA). **(D)** Resorption pit formation on bone slices (Scale bars: 50 μm). **(E)** Immunofluorescence staining of F-actin rings (Scale bars: 100 μm). **(F)** Quantification of actin ring formation. The data were shown as the mean ± SD (*n* = 6); ∗*P* < 0.05; ∗∗*P* < 0.01; ∗∗∗*P* < 0.001 indicated significant differences between the indicated columns (one-way ANOVA). **(G)** Fluorescence imaging of pre-osteoclast fusion labeled with Dil and Hoechst after 24 h co-culture (Scale bars: 100 μm). **(H)** Quantification of fused cells. The data were shown as the mean ± SD (*n* = 6); ∗*P* < 0.05; ∗∗*P* < 0.01; ∗∗∗*P* < 0.001 indicated significant differences between the indicated columns (one-way ANOVA). **(I)** Flow cytometry analysis of CD11b, CD51, RANK, and PI in osteoclasts. **(J)** PI intensity analysis of CD11b^+^ CD51^+^ RANK^+^ populations. **(K)** qRT-PCR analysis of *Pdgf-bb* mRNA expression. **(L)** ELISA quantification of PDGF-BB protein secretion. The data were shown as the mean ± SD (*n* = 6); ∗*P* < 0.05; ∗∗*P* < 0.01; ∗∗∗*P* < 0.001 indicated significant differences between the indicated points (one-way ANOVA).

To evaluate the regulatory effects of different pore-sized Mg-Ti composites on osteoclast differentiation, we established an *in vitro* osteoclast differentiation model based on mouse Bone Marrow Monocytes/Macrophages (BMMs) (Fig. 4A). Under Macrophage Colony-Stimulating Factor (M-CSF) and Receptor Activator of Nuclear Factor Kappa-B Ligand (RANKL) co-induction conditions, different group extracts exhibited differential osteoclast regulatory effects.

To systematically characterize the impact of surface modifications on osteoclastogenesis, we performed sequential functional analyses spanning differentiation through mature bone resorption activity. Tartrate-Resistant Acid Phosphatase (TRAP) staining was employed to evaluate osteoclast differentiation, utilizing TRAP as a definitive enzymatic marker of the osteoclast lineage. While pure Ti demonstrated negligible inhibitory effects, Mg-Ti-900 exhibited modest suppression relative to Control. Conversely, both Mg-Ti-1200 and Mg-Ti-1500 induced statistically significant and equivalent inhibition of osteoclast differentiation (Fig. 4B and C). Corroborative experiments utilizing RAW 264.7 cells, a well-established model for *in vitro* osteoclastogenesis [39], demonstrated congruent inhibitory patterns across all treatment groups (Fig. S13A). SEM analysis confirmed the established inhibitory hierarchy: Mg-Ti-900 induced modest resorption suppression, whereas Mg-Ti-1200 and Mg-Ti-1500 demonstrated profound and statistically equivalent inhibition of osteoclastic bone resorption capacity (Fig. 4D).

Given that multinucleated giant cell formation constituted an obligatory prerequisite for functional osteoclast development, cell fusion kinetics were subsequently examined. Actin ring immunofluorescence, which served as a definitive marker of osteoclast polarization and maturation, corroborated these findings, demonstrating that Mg-Ti-1200 and Mg-Ti-1500 significantly disrupted characteristic peripheral actin ring architecture compared to Mg-Ti-900 (Fig. 4E and F). Cell fusion quantitative analysis revealed dose-dependent inhibition, with Mg-Ti-900 producing marginal effects while Mg-Ti-1200 and Mg-Ti-1500 demonstrated substantially enhanced and statistically equivalent suppression of cell fusion (Fig. 4G and H). Functional validation was achieved through bone resorption assays utilizing differentiated BMMs cultured on bovine bone substrates. Immunofluorescence analysis of CTSK, a critical cysteine protease essential for extracellular matrix degradation, exhibited consistent inhibitory trends across experimental conditions (Fig. S13B), thereby validating the functional impairment observed in bone resorption analyses. These results indicated that in terms of osteoclast differentiation and functional inhibition, Mg-Ti-1200 and Mg-Ti-1500 demonstrated similar effects, both significantly superior to Mg-Ti-900. Transcriptional-level analysis confirmed the above functional observations. qRT-PCR detection of osteoclast-specific genes *Ocstamp*, *Dcstamp*, *Ctsk* and *Mmp9* expression showed that Mg-Ti-1200 and Mg-Ti-1500 significantly downregulated these key differentiation and functional genes (Fig. S13C), validating the comparable inhibitory effects at the molecular level.

Although Mg-Ti-1200 and Mg-Ti-1500 showed similar osteoclast inhibition, flow cytometry revealed key differences. CD11b^+^ cell gating analysis of CD51 and RANK expression demonstrated that both groups significantly increased CD11b^+^ CD51^−^ RANK^+^ osteoclast precursor cells (Q3) (Fig. 4I), confirming effective arrest of differentiation at the precursor stage. However, apoptosis analysis showed that Mg-Ti-1500 precursors exhibited significantly elevated PI-positive rates (Fig. 4J), indicating toxic effects from excessive Mg ions, while Mg-Ti-1200 maintained low apoptosis levels. This survival difference directly affected precursor secretory function. Since osteoclast precursors secrete PDGF-BB [[19], [20], [21]], a key bone repair coupling factor, we examined PDGF-BB expression and secretion across groups. qRT-PCR showed highest *Pdgf-bb* expression in Mg-Ti-1200 (Fig. 4K), which Enzyme-Linked Immunosorbent Assay (ELISA) confirmed at the protein level with strongest PDGF-BB release (Fig. 4I). Despite maintaining precursor cell numbers, Mg-Ti-1500 showed markedly reduced PDGF-BB secretion due to compromised cellular function, underscoring the importance of cell viability for functional maintenance.

### 2.5 PDGF-BB-mediated coordination of osteogenesis and angiogenesis

**Fig. 5:**
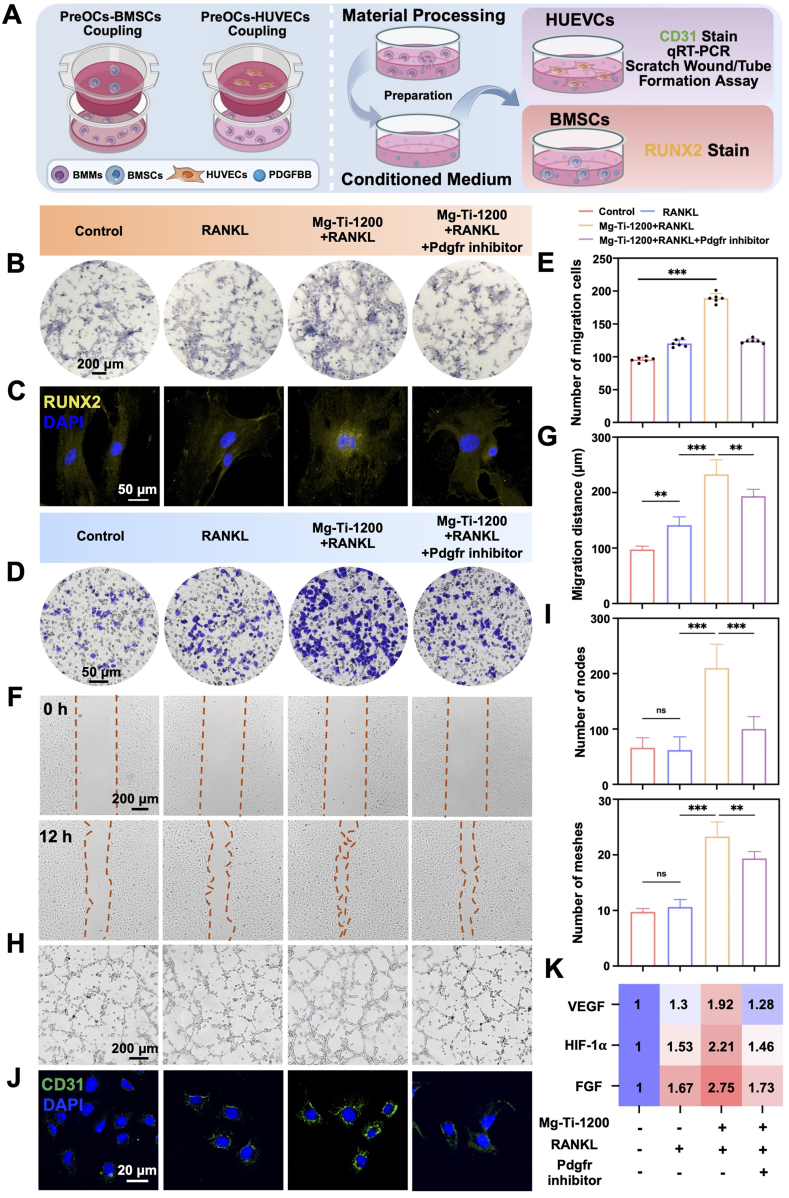
**Modulation of osteogenesis and angiogenesis by Mg-Ti composites via PDGF-BB: (A)** Schematic illustration depicting osteoclast precursors-derived PDGF-BB-mediated coupling of osteogenesis and angiogenesis. **(B)** ALP staining of BMSCs in a Transwell co-culture system (Scale bars: 200 μm). **(C)** Immunofluorescence analysis of RUNX2 expression in BMSCs (Scale bars: 50 μm). **(D)** Representative images of HUVECs migration assay (Scale bars: 500 μm). **(E)** Quantification of HUVECs migratory capacity. The data were shown as the mean ± SD (*n* = 6); ∗*P* < 0.05; ∗∗*P* < 0.01; ∗∗∗*P* < 0.001 indicated significant differences between the indicated columns (one-way ANOVA). **(F)** Scratch wound healing assay assessing HUVECs migration at 0 and 12 h (Scale bars: 200 μm). **(G)** Quantification of scratch wound closure area. The data were shown as the mean ± SD (*n* = 6); ∗*P* < 0.05; ∗∗*P* < 0.01; ∗∗∗*P* < 0.001 indicated significant differences between the indicated columns (one-way ANOVA). **(H)** Tube formation assay of HUVECs cultured with conditioned media (Scale bars: 200 μm). **(I)** Quantitative analysis of capillary-like network formation. The data were shown as the mean ± SD (*n* = 6); ∗*P* < 0.05; ∗∗*P* < 0.01; ∗∗∗*P* < 0.001 indicated significant differences between the indicated columns (one-way ANOVA). **(J)** Immunofluorescence staining of CD31 in HUVECs post-treatment (Scale bars: 20 μm). **(K)** qRT-PCR analysis of angiogenic (*VEGF*, *FGF*, *HIF-1*α) gene expression in HUVECs.

Given that Mg-Ti-1200 exhibited the most significant PDGF-BB secretion capacity, we further validated whether this coupling factor could mediate coordinated regulation of osteogenesis and vascularization. We constructed a Transwell co-culture system with experimental designs including Control, RANKL, Mg-Ti-1200 extract combined with RANKL, and Mg-Ti-1200 extract combined with RANKL plus PDGFR inhibitor to determine the specific role of PDGF-BB in bone-vascular coupling [40]. In all groups, M-CSF was added by default (Fig. 5A).

Osteogenic coupling validation showed the Mg-Ti-1200 + RANKL group had strongest pro-differentiation activity. In BMSCs-osteoclast co-culture systems, Mg-Ti-1200 + RANKL treatment of lower chamber cells significantly enhanced upper chamber BMSCs ALP activity (Fig. 5B). Immunofluorescence confirmed that Mg-Ti-1200 + RANKL conditioned medium induced strongest RUNX2 expression and nuclear translocation (Fig. 5C), indicating effective osteogenic transcriptional activation. Critically, PDGFR inhibitor addition significantly attenuated this effect, confirming PDGF-BB necessity.

Angiogenic analysis confirmed Mg-Ti-1200 + RANKL pro-angiogenic activity. Transwell assays showed its conditioned medium significantly promoted Human Umbilical Vein Endothelial Cells (HUVECs) migration, with markedly increased cell numbers versus other groups (Fig. 5D and E). Scratch assays validated this pro-migratory effect, with accelerated endothelial monolayer repair (Fig. 5F and G). Tube formation assays demonstrated the most complete tubular networks, superior in both number and connectivity (Fig. 5H and I). Endothelial phenotype analysis supported these angiogenic effects. CD31 immunofluorescence showed HUVECs treated with Mg-Ti-1200 + RANKL exhibited strongest endothelial marker expression with typical morphological characteristics (Fig. 5J). qRT-PCR heatmaps confirmed strongest upregulation of angiogenesis-related genes in the Mg-Ti-1200 + RANKL group, while Platelet-Derived Growth Factor Receptor (PDGFR) inhibitor significantly reversed this transcriptional activation (Fig. 5K), confirming PDGF-BB-mediated regulation at the molecular level.

These findings demonstrated that Mg-Ti-1200 arrested osteoclast differentiation, arresting cells at the preosteoclast stage where they adopt a coupling-promoting phenotype characterized by enhanced PDGF-BB secretion rather than progressing to mature bone-resorbing osteoclasts. This phenotypic shift redirected cellular function from destructive bone resorption toward constructive paracrine signaling that simultaneously promotes osteogenesis and angiogenesis. The PDGF-BB-mediated coupling mechanism was particularly significant for load-bearing bone defect repair, as it enabled synchronized bone formation with vascular network establishment, critical for restoring mechanical strength and ensuring adequate nutrient supply in weight-bearing applications. This approach transformed the traditionally detrimental osteoclastic response into a therapeutically beneficial process that accelerated bone regeneration while maintaining structural integrity essential for load-bearing bone defects.

### 2.6 Molecular mechanisms of Mg-Ti-1200 extract-mediated osteoclast inhibition

**Fig. 6:**
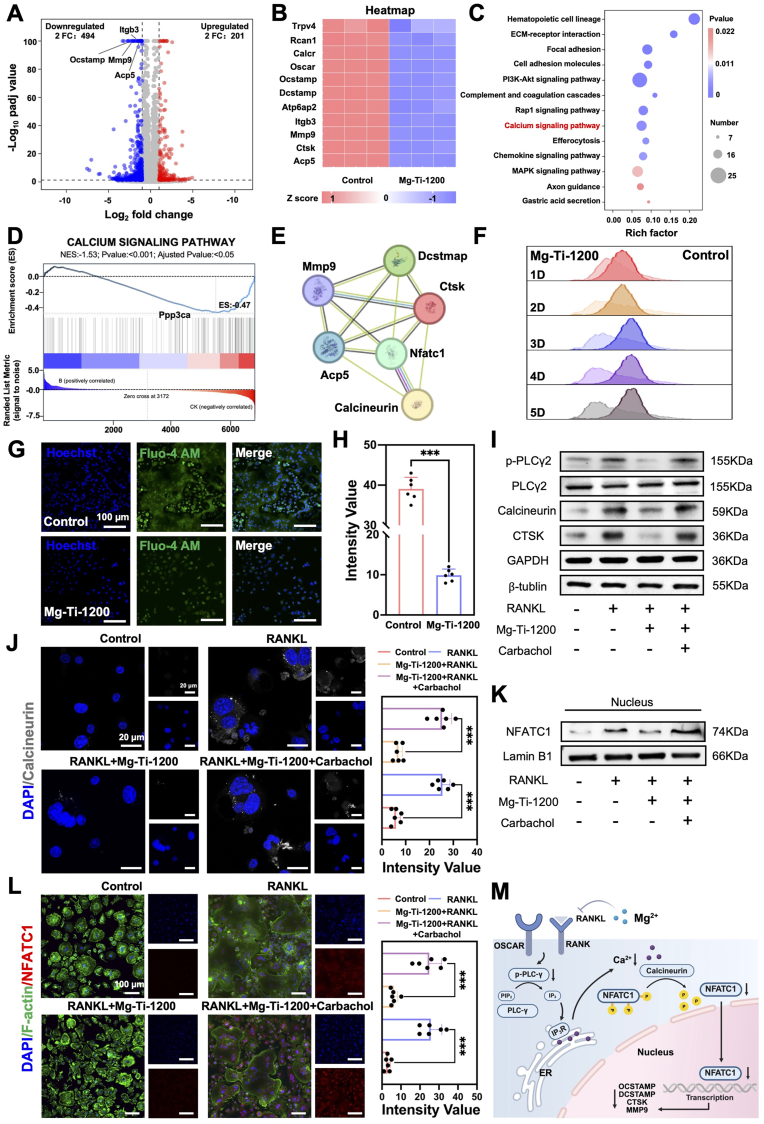
**Mechanistic analysis of osteoclast differentiation inhibition by Mg-Ti-1200:** (A) Volcano plot showing differential gene expression between Mg-Ti-1200 and Control. (B) Osteoclast-related gene expression changes upon Mg-Ti-1200 treatment compared to Control. (C) KEGG pathway analysis between Mg-Ti-1200 and Control. (D) Gene set enrichment analysis of calcium ion signaling pathways, highlighting downregulation of *Ppp3ca*. (E) PPI network showing interaction between calcineurin (encoded by *Ppp3ca*) and NFATc1, regulating osteoclast-related genes. (F) Flow cytometry analysis of intracellular calcium levels in osteoclasts Within 5 days. (G) Fluorescence imaging of intracellular calcium levels (Fluo-4 staining) (Scale bars: 100 μm). (H) Quantification of intracellular calcium concentrations from Fluo-4 staining. The data were shown as the mean ± SD (*n* = 6); ∗*P* < 0.05; ∗∗*P* < 0.01; ∗∗∗*P* < 0.001 indicated significant differences between the indicated columns (*t*-test). (I) Western blot of PLCγ2-Calcineurin-NFATc1 signaling proteins in different treatments. (J) Immunofluorescence of calcineurin (Scale bars: low magnification: 20 μm, high magnification: 20 μm). The data were shown as the mean ± SD (*n* = 6); ∗*P* < 0.05; ∗∗*P* < 0.01; ∗∗∗*P* < 0.001 indicated significant differences between the indicated columns (one-way ANOVA). (K) Western blot analysis of NFATc1 nuclear translocation. (L) Immunofluorescence of NFATc1 (Scale bars: low magnification: 100 μm, high magnification: 100 μm). The data were shown as the mean ± SD (*n* = 6); ∗*P* < 0.05; ∗∗*P* < 0.01; ∗∗∗*P* < 0.001 indicated significant differences between the indicated columns (one-way ANOVA). (M) Schematic summarizing the signaling cascade.

To elucidate the molecular mechanisms by which Mg-Ti-1200 extract inhibited osteoclast differentiation, we performed transcriptomic analysis on osteoclast-induced cells after treatment. Differential gene expression analysis showed that under the stringent threshold (Fold Change = 2), Mg-Ti-1200 treatment caused downregulation of 494 genes and upregulation of 201 genes, presenting an obvious transcriptional suppression pattern (Fig. 6A). Key osteoclast functional genes including matrix degrading enzyme *Mmp9*, cell fusion molecule *Ocstamp*, integrin *Itgb3*, and acid phosphatase *Acp5* all showed significant downregulation. Clustering heatmaps confirmed systematic inhibition of core osteoclast differentiation gene networks, encompassing coordinated downregulation of cell fusion, bone resorption, and osteoclast-specific marker genes (Fig. 6B). Pathway enrichment analysis revealed upstream regulatory mechanisms of transcriptional suppression. KEGG analysis identified significant alterations in calcium signaling pathway (Fig. 6C), pointing to key regulatory nodes in osteoclast differentiation. Gene Set Enrichment Analysis (GSEA) further confirmed overall downregulation trends in calcium signaling pathways and specifically identified significant reduction in *Ppp3ca* expression (Fig. 6D). *Ppp3ca* encodes the catalytic subunit of calcineurin, a calcium-dependent serine/threonine phosphatase that regulates immune responses and calcium signaling pathway [[41], [42], [43]]. Protein-Protein Interaction (PPI) network analysis showed direct interactions between calcineurin and NFATc1, occupying central node positions in osteoclast differentiation-related protein networks (Fig. 6E), suggesting its critical role in osteoclast differentiation regulation. *Ppp3ca* encoded the calcineurin catalytic subunit, an essential enzyme for NFATc1 dephosphorylation and activation, and its downregulation suggested that Mg-Ti-1200 might block osteoclast differentiation by interfering with the Calcium-Calcineurin-NFATc1 axis.

Based on transcriptomic predictions, we validated Mg-Ti-1200's regulatory effects on calcium ion homeostasis. Dynamic monitoring showed that Mg-Ti-1200 treatment continuously suppressed intracellular calcium ion concentrations throughout the entire 5-day differentiation period (Fig. 6F). End-point detection with Fluo-4 fluorescence staining further confirmed that Mg-Ti-1200 cells exhibited significantly lower calcium ion fluorescence intensity compared to Control (Fig. 6G and H), establishing direct correlations between transcriptional changes and functional phenotypes.

Protein-level validation confirmed calcium signaling pathway inhibition mechanisms. In osteoclast differentiation, PLCγ2 underwent tyrosine phosphorylation upon RANKL stimulation and catalyzed PIP2 hydrolysis to generate IP3 and DAG, triggering intracellular calcium mobilization essential for osteoclastogenesis [[43], [44], [45]]. This calcium signaling activated the PLCγ2-Calcineurin-NFATc1 pathway, where calcium-activated calcineurin (encoded by *Ppp3ca*) dephosphorylated NFATc1, promoting its nuclear translocation and transcriptional activation of osteoclast-specific genes like *Ocstamp*, *Dcstamp*, *Ctsk*, and *Mmp9*, which respectively mediated osteoclast precursors fusion and bone resorption functions. Western blot analysis of the PLCγ2-Calcineurin-NFATc1 signaling axis demonstrated that RANKL stimulation significantly upregulated PLCγ2 phosphorylation, calcineurin expression and the osteoclast marker CTSK, which were markedly attenuated by Mg-Ti-1200 extract. We employed carbachol as a pathway agonist in rescue experiments and found it effectively reversed Mg-Ti-1200-mediated inhibition and reactivated the pathway (Fig. 6I). Immunofluorescence imaging confirmed that Mg-Ti-1200 treatment substantially reduced cytoplasmic calcineurin fluorescence, which was restored by carbachol co-treatment (Fig. 6J). Nuclear protein fractionation showed that RANKL promoted NFATc1 nuclear translocation, Mg-Ti-1200 inhibited this process, while carbachol treatment reconstituted nuclear translocation (Fig. 6K). Quantitative immunofluorescence analysis confirmed that Mg-Ti-1200 significantly decreased NFATc1 nuclear fluorescence intensity and nuclear-to-cytoplasmic ratios, effects that were largely reversed by carbachol (Fig. 6L), demonstrating the precise reversibility of this signaling pathway regulation. M-CSF was supplemented in all groups to maintain cell viability.

These mechanistic findings provided a molecular basis for understanding why Mg-Ti-1200-treated cells remained arrested at the osteoclast precursors stage while exhibiting enhanced PDGF-BB secretion. The disruption of the PLCγ2-Calcineurin-NFATc1 signaling axis prevented transcriptional activation of terminal osteoclast differentiation genes such as *Ocstamp*, *Dcstamp*, and *Ctsk*, which were essential for multinucleation and bone resorption function (Fig. 6M). However, this differentiation arrest did not compromise the cells’ capacity for paracrine factor production. Instead, by blocking progression to the energy-intensive mature osteoclast phenotype, cellular resources were redirected toward secretory functions, particularly the production of coupling factors like PDGF-BB. This metabolic and functional arresting represented an adaptive response wherein osteoclast precursors, unabled to complete terminal differentiation due to impaired NFATc1 signaling, adopted an alternative phenotype focused on tissue repair coordination rather than bone destruction.

### 2.7 *In vivo* bone regeneration assessment

**Fig. 7:**
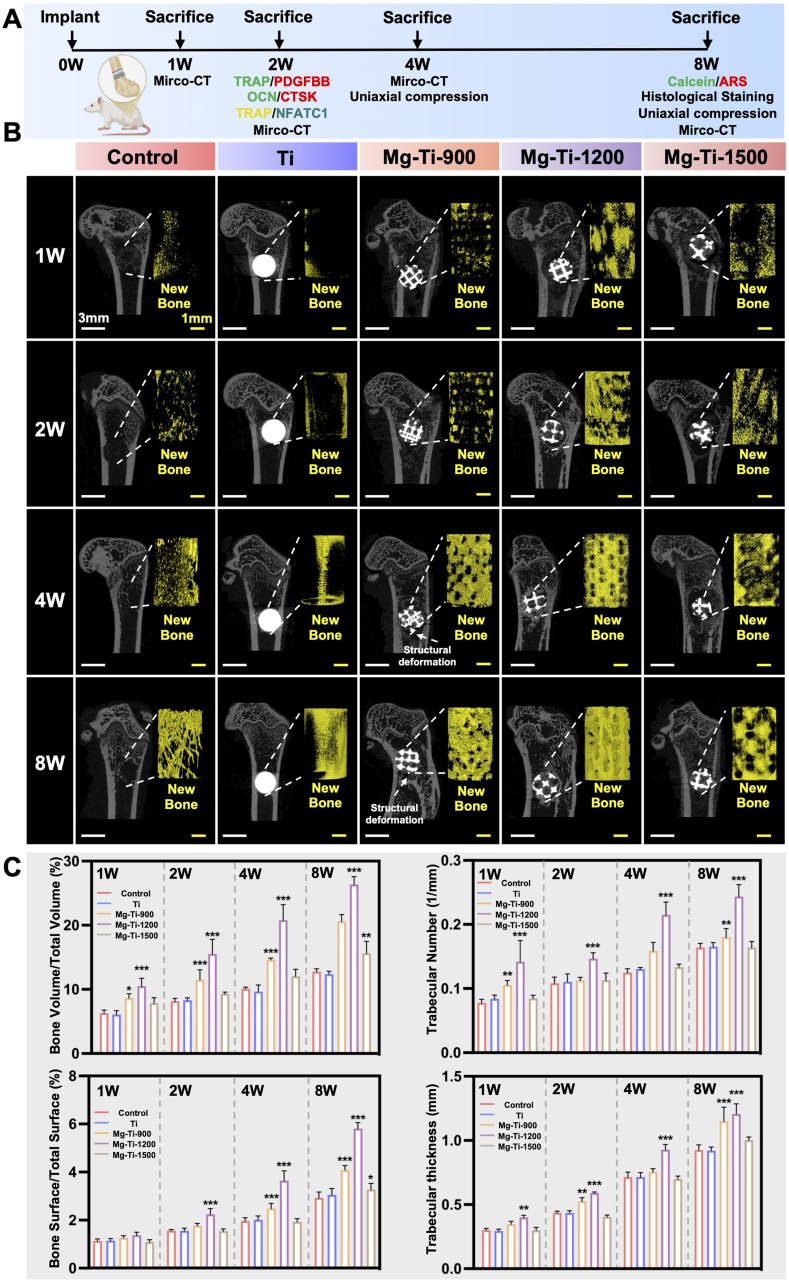
***In vivo* evaluation of osteogenesis induced by Mg-Ti composites:** (A) Schematic representation of the experimental design. (B) Representative micro-CT images of femur sections at 1-, 2-, 4-, and 8-weeks post-implantation for each material group (Control, Ti, Mg-Ti-900, Mg-Ti-1200, Mg-Ti-1500) (Scale bars: low magnification: 3 mm, high magnification: 1 mm). New bone formation is highlighted in yellow. (C) Quantitative analysis of bone parameters at each time point: bone volume/total volume (BV/TV), bone surface/total surface (BS/TS), trabecular number (Tb.N), and trabecular thickness (Tb.Th) for each treatment group. The data were shown as the mean ± SD (*n* = 6); ∗*P* < 0.05; ∗∗*P* < 0.01; ∗∗∗*P* < 0.001 indicated significant differences between the indicated columns (two-way ANOVA).

To validate the *in vivo* bone regenerative capacity of different pore-sized Mg-Ti composites, we established a rat femoral defect model and evaluated bone regeneration effects through micro-CT analysis and histological analysis (Fig. 7A). Safety assessment indicated that all tested materials exhibited excellent biosafety and hemocompatibility *in vivo* (Fig. S14). Three-dimensional reconstruction images showed significant differences among groups in new bone formation and scaffold integration (Fig. 7B). Control and pure Ti groups showed limited new bone formation with relatively low bone tissue regeneration in defect regions. Mg-Ti-900 induced moderate bone regenerative responses with new bone tissue penetrating scaffold pores, but bone ingrowth depth was limited. Mg-Ti-1200 induced the most significant bone regenerative response, with new bone tissue marked penetrating the internal pore network of scaffolds, forming continuous bone tissue architecture and achieving good integration at scaffold-host bone interfaces. Mg-Ti-1500 promoted new bone formation but scaffold internal bone ingrowth was significantly lower than Mg-Ti-1200. Quantitative analysis further confirmed Mg-Ti-1200's superior performance (Fig. 7C). Bone volume fraction (BV/TV) analysis showed that Mg-Ti-1200 exhibited the highest values at all time points, significantly exceeding other groups. Trabecular number (Tb.N) analysis indicated that Mg-Ti-1200 formed the densest trabecular bone network structure, reflecting its advantages in bone tissue microarchitectural reconstruction. Bone surface area to total surface ratio (BS/TS) results showed that Mg-Ti-1200 had the largest active bone formation surface, indicating sustained bone remodeling activity. Trabecular thickness (Tb.Th) analysis confirmed that Mg-Ti-1200 formed the most robust trabecular structures, indicating optimized bone quality. These morphometric parameters consistently demonstrated that Mg-Ti-1200 achieved optimal levels in bone regeneration quantity, quality, and microstructural reconstruction. To further validate the biological mechanisms underlying Mg-Ti-1200 superiority, we performed cellular and molecular-level analyses of post-implantation tissues.

**Fig. 8:**
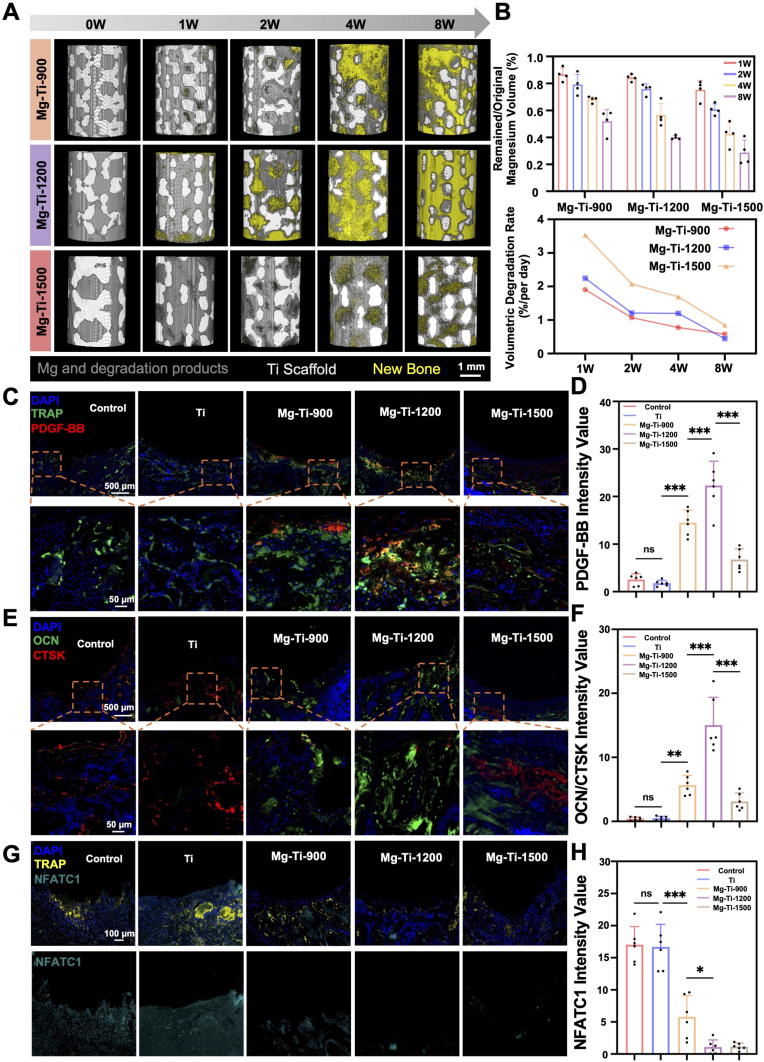
***In vivo* assessment of material degradation and osteogenesis/osteoclastogenesis:** (A) 3D reconstructions depicting the degradation of Mg-Ti composites (Mg-Ti-900, Mg-Ti-1200, Mg-Ti-1500) and new bone formation (yellow) (Scale bars: 1 mm). (B) Quantification of residual magnesium phase and degradation rate for each composite. (C) Immunofluorescence staining for TRAP (red) and PDGF-BB (green), highlighting osteoclast activity and PDGF-BB expression at week 2 (Scale bars: low magnification: 500 μm, high magnification: 50 μm). (D) Quantification of PDGF-BB fluorescence intensity. The data were shown as the mean ± SD (*n* = 6); ∗*P* < 0.05; ∗∗*P* < 0.01; ∗∗∗*P* < 0.001 indicated significant differences between the indicated columns (one-way ANOVA). (E) Dual immunofluorescence staining for OCN (red) and CTSK (green), evaluating osteoblast and osteoclast activity at week 2 (Scale bars: low magnification: 500 μm, high magnification: 50 μm). (F) Quantification of the OCN/CTSK ratio. The data were shown as the mean ± SD (*n* = 6); ∗*P* < 0.05; ∗∗*P* < 0.01; ∗∗∗*P* < 0.001 indicated significant differences between the indicated columns (one-way ANOVA). (G) Immunofluorescence staining for TRAP (red) and NFATc1 (green) at week 2 (Scale bars: 100 μm). (H) Quantification of NFATc1 fluorescence intensity. The data were shown as the mean ± SD (*n* = 6); ∗*P* < 0.05; ∗∗*P* < 0.01; ∗∗∗*P* < 0.001 indicated significant differences between the indicated columns (one-way ANOVA).

Building upon the superior *in vitro* performance of Mg-Ti-1200 in maintaining osteoclast precursors and promoting PDGF-BB secretion, we hypothesized that these biological effects would be closely linked to the *in vivo* degradation kinetics of the composites. To test this and establish structure-function relationships, we assessed the degradation behavior and biological responses of Mg-Ti composites in a rat femoral defect model. Three-dimensional reconstruction of the Mg phase showed pore size-dependent degradation kinetics that aligned with *in vitro* activities: Mg-Ti-900 displayed slow degradation, Mg-Ti-1500 showed accelerated degradation, and Mg-Ti-1200 exhibited moderate, controlled rates (Fig. 8A). Quantitative analysis of residual Mg volume at 8 weeks revealed retention rates of approximately 52.1 ± 1.2 % for Mg-Ti-900, 40.4 ± 2.2 % for Mg-Ti-1200, and 25.6 ± 1.7 % for Mg-Ti-1500 (Fig. 8B). Temporal degradation rates varied distinctly: Mg-Ti-900 decreased from 2.1 %/day initially to <0.8 %/day later; Mg-Ti-1200 showed a controlled decline from 2.3 %/day to 0.7 %/day; and Mg-Ti-1500 maintained high rates, dropping from >3.5 %/day to 1.1 %/day (Fig. 8B). These patterns indicated that Mg-Ti-1200's optimal *in vitro* performance corresponds to its balanced degradation, likely enabling sustained, controlled release of bioactive ions for osteoclast arresting.

PDGF-BB expression in the implantation region confirmed this degradation-dependent response, mirroring *in vitro* co-culture results. Immunofluorescence at 2 weeks showed no significant PDGF-BB signals in Control and pure Ti groups (Fig. 8C and D). Mg-Ti-900, with slow degradation, displayed limited expression at the material-tissue interface, reflecting insufficient ionic levels for optimal osteoclast precursor maintenance. Mg-Ti-1200, with balanced degradation, induced the most extensive and intense PDGF-BB expression, with fluorescence intensity surpassing other groups, validating its *in vitro* superiority in maintaining osteoclast precursor function. Although Mg-Ti-1500 induced PDGF-BB expression, its intensity and distribution were notably lower than Mg-Ti-1200, consistent with *in vitro* findings of excessive ionic stress causing increased apoptosis and impaired secretion.

Histological analysis of bone remodeling revealed tissue responses to varying degradation environments. OCN/CTSK dual immunofluorescence at 2 weeks indicated that Mg-Ti-1200 tissues showed the strongest osteogenic-dominant phenotype, with extensive OCN expression (active bone formation) and suppressed CTSK expression (reduced resorption) (Fig. 8E). Control and pure Ti groups were CTSK-dominant with limited OCN. Mg-Ti-900 showed moderate osteogenic enhancement, while Mg-Ti-1500's was inferior to Mg-Ti-1200. Quantitative OCN/CTSK ratios confirmed Mg-Ti-1200's advantage in modulating osteogenic/osteoclastic activity (Fig. 8F).

To confirm *in vitro* mechanistic findings on Calcium-Calcineurin-NFATc1 pathway inhibition *in vivo*, NFATc1 subcellular localization was analyzed at 2 weeks. TRAP/NFATc1 dual immunofluorescence revealed strong nuclear NFATc1 in Control and pure Ti osteoclasts (Fig. 8G). Consistent with *in vitro* results, Mg-Ti-1200 and Mg-Ti-1500 showed comparable inhibition of NFATc1 nuclear translocation, with predominant cytoplasmic localization (Fig. 8H), validating the *in vivo* relevance of PLCγ2-Calcineurin-NFATc1 signaling inhibition.

*In vivo* validation experiments corroborated the core *in vitro* mechanistic findings, demonstrating that the Mg ionic microenvironment served as the primary driver of early tissue remodeling with pronounced osteogenic bias. While both Mg-Ti-1200 and Mg-Ti-1500 exhibited comparable osteoclast differentiation inhibition through NFATc1 pathway suppression, Mg-Ti-1200's balanced degradation kinetics established optimal ionic gradients that facilitated early microenvironmental transition toward osteogenic predominance, as evidenced by enhanced OCN/CTSK expression ratios during critical early implantation periods. The concurrent enhancement of PDGF-BB expression within Mg-Ti-1200 tissues suggested synergistic coupling mechanisms that amplified both osteogenic and angiogenic processes beyond primary ionic effects, contributing to integrated bone-vascular network establishment essential for sustained osseointegration. Collectively, these findings demonstrated that the controlled degradation profile of Mg-Ti-1200 represented an optimal biomaterial design approach, achieving superior bone regeneration outcomes through coordinated modulation of cellular differentiation pathways and establishment of early osteogenic-dominant microenvironments critical for load-bearing implant applications.

### 2.8 Histomorphometric and biomechanical characterization

**Fig. 9:**
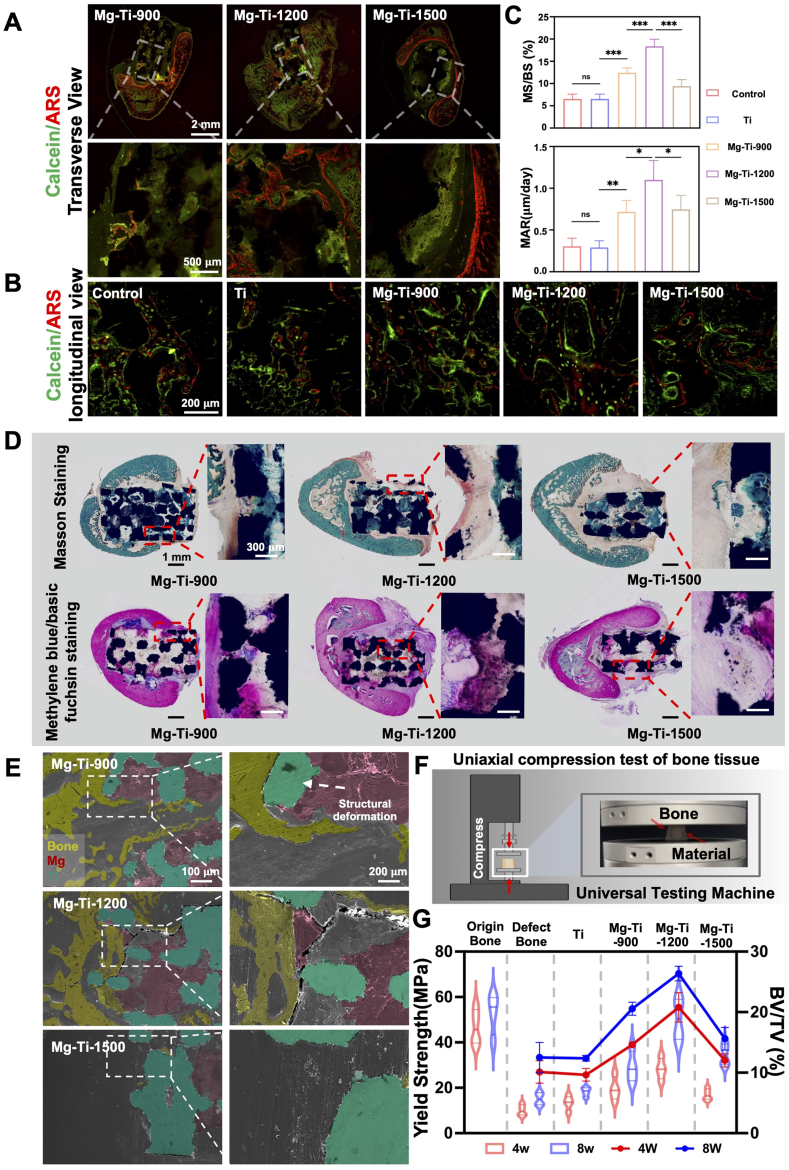
***In vivo* evaluation of bone growth, mineralization, and mechanical properties:** (A, B) Representative images of Calcein (green) and Alizarin Red (red) labeling in transverse (Scale bars: low magnification: 2 mm, high magnification: 500 μm) and longitudinal (Scale bars: 200 μm) sections of the implants. (C) Quantitative analysis of mineralized surface/total bone surface (MS/BS) and mineral apposition rate (MAR). The data were shown as the mean ± SD (*n* = 6); ∗*P* < 0.05; ∗∗*P* < 0.01; ∗∗∗*P* < 0.001 indicated significant differences between the indicated columns (one-way ANOVA). (D) Masson's trichrome and Methylene Blue/Fuchsin staining (Scale bars: low magnification: 1 mm, high magnification: 300 μm). (E) SEM images of bone tissue cross-sections, highlighting newly formed bone (yellow), residual Mg phase (red), and pure Ti scaffold (cyan) (Scale bars: low magnification: 100 μm, high magnification: 200 μm). (F) Uniaxial compression test of bone tissue. (G) Quantification of yield strength (MPa) for different materials in bone tissue (*n* = 4) at 4 and 8 weeks.

To assess the histological efficacy of Mg-Ti composites in promoting osseointegration and bone regeneration, we conducted fluorochrome labeling and histomorphometric analysis of mineralized tissue post-implantation. Calcein Green and Alizarin Red were used to label newly formed and mineralized bone, respectively, enabling assessment of bone formation dynamics. Histomorphometric analysis of longitudinal sections revealed distinct intergroup differences in osseous tissue penetration and mineralization across scaffold architectures (Fig. 9A). Mg-Ti-900 exhibited moderate osteoconductivity with visible bone tissue infiltration, indicated by Calcein Green labeling, but limited mineralization as shown by weak Alizarin Red incorporation. Mg-Ti-1200 demonstrated superior osteointegration, with extensive bone penetration into scaffold pores and robust mineralization, evidenced by abundant Calcein Green and Alizarin Red deposition. In contrast, Mg-Ti-1500 showed restricted osseointegration, mostly confined to the scaffold periphery, with minimal fluorochrome labeling. High-magnification images further confirmed these differences (Fig. 9A). Mg-Ti-1200 exhibited high-density Calcein Green and Alizarin Red labeling within scaffold interiors, indicating vigorous osteogenesis and advanced mineralization. Mg-Ti-900 showed moderate osteogenesis but limited mineralization, while Mg-Ti-1500 showed minimal fluorochrome incorporation, confirming its poor osteoconductive and osteoinductive capacity. Longitudinal section growth line analysis quantified mineralization kinetics, with Mg-Ti-1200 displaying optimal growth line spacing, while Mg-Ti-900 had moderate deposition, and Mg-Ti-1500 showed compressed growth lines, particularly at the scaffold periphery (Fig. 9B). Mineral apposition rate (MAR) analysis confirmed these findings, with Mg-Ti-1200 showing significantly higher MAR values than the other groups (Fig. 9C). Histological evaluation with Masson's trichrome and Methylene Blue-Phloxine staining further highlighted differences in bone tissue organization (Fig. 9D). Mg-Ti-1200 induced abundant collagen matrix formation and mature, well-organized trabecular bone. Mg-Ti-900 showed moderate collagen deposition with scattered bone formation, while Mg-Ti-1500 had limited collagen and immature tissue structure. These differences were consistent with the fluorochrome labeling results, confirming Mg-Ti-1200's superior bone regeneration capacity. SEM of the bone-implant interface revealed distinct tissue responses across different pore sizes (Fig. 9E). The Mg-Ti-900 group showed suboptimal ionic release and localized structural collapse, hindering bone ingrowth. Mg-Ti-1500 exhibited excessive Mg dissolution, disrupting osteoclast and osteoblast activity. Mg-Ti-1200 demonstrated optimal ionic release, arresting osteoclast differentiation while promoting osteoblast activity, maintaining scaffold integrity and enabling efficient bone formation.

Compressive mechanical testing at 4 and 8 weeks post-implantation demonstrated that Mg-Ti-1200 achieved compressive strengths comparable to native bone, with marked improvement by 8 weeks (Fig. 9G). This underscored Mg-Ti-1200's exceptional osseointegration and structural resilience in load-bearing conditions. Finite Element Analysis (FEA) at 8 weeks (Fig. S15) revealed optimal stress distribution at the bone-implant interface, mitigating stress shielding and averting localized stress concentrations observed in Mg-Ti-900 and Mg-Ti-1500. Compared to 3D-printed non-degradable scaffolds, such as porous tantalum and surface-modified Ti, Mg-Ti-1200 exhibits superior bioactivity and controlled degradability, while polymer-based scaffolds lack sufficient mechanical robustness for load-bearing applications. Alternative biodegradable metals, such as zinc or iron within titanium matrices, may offer comparable biomechanical benefits, albeit with distinct degradation kinetics. While the 8-week small-animal model provides robust preliminary insights, future studies in large-animal models and clinical settings are warranted to further elucidate long-term degradation dynamics and systemic biocompatibility.

## 3 Conclusion

Guided by scRNA-seq profiling, this study identified the dual regulatory role of Mg ions in bone regeneration and leveraged this insight to develop Mg-Ti composites capable of intelligently modulating the Osteogenesis and Osteoclastogenesis. Mechanistic investigations within the material system revealed that Mg ions promoted osteogenesis via PI3K-Akt signaling activation, while simultaneously suppressing terminal osteoclast differentiation by inhibiting the PLCγ2-Calcineurin-NFATc1 axis. Importantly, this strategy preserved PDGF-BB secretion from osteoclast precursors, enabling coordinated regulation of osteogenesis and angiogenesis: an “arresting” approached that transcends traditional “pro-osteogenic, anti-osteoclastic” paradigms. Among the designs, Mg-Ti-1200 composites achieved optimal Mg ions release through precise pore architecture, exhibiting robust pro-osteogenic, osteoclast-modulating, and pro-angiogenic performance. *In vivo* evaluation confirmed significant enhancement of load-bearing bone defect repair, along with favorable degradation behavior and biomechanical compatibility. This study introduced a dual strategy of “compositional evolution” and “mechanism-informed design,” establishing a translational framework from single-cell-guided biological insight to functional material innovation. These findings provided a compelling foundation for the next generation of bone repair materials with strong clinical and engineering potential.

## 4 Experimental section

*Preparation of Ti and Mg Implants for scRNA-seq:* Ti and Mg rods (Φ1 mm × 2 mm) were polished and sterilized with ethylene oxide. A femoral defect (Φ1 mm × 2 mm) was created in 6-week-old male C57BL/6J mice (*n* = 6 per group) under anesthesia. Implants were inserted into the defect sites, and incisions were closed with 6-0 nylon sutures. On day 7 post-surgery, peri-implant tissues were collected for scRNA-seq (Personal Biotechnology Co., Ltd. Shanghai, China). All procedures were approved by the Institutional Animal Care and Use Committee of China Medical University (Project Number: CMU20251305)

*Fabrication of Mg-Ti Composites:* Porous Ti scaffolds (Φ11 mm × 20 mm) with rhombic dodecahedral architecture were fabricated via Selective Laser Melting (SLM) using Ti powders (15–53 μm). The pure Mg blocks were placed on top of the 3D printed Ti scaffolds in a high-purity graphite crucible. The materials were heated in flowing argon gas at a heating rate of 10 °C/min to 850 °C (about 200 °C above the melting point of Mg), held for 1 h, and then slowly cooled in the furnace. Prior to infiltration, the Mg blocks were mechanically polished and then ultrasonically cleaned along with the scaffolds in acetone to reduce contamination. Pure Ti served as Control. For *in vitro* use, samples (Φ10 mm × 2 mm) were polished, ultrasonicated in acetone and ethanol, and UV-sterilized. For *in vivo* use, samples (Φ3 mm × 5 mm) were polished and sterilized with ethylene oxide.

*Microstructural Characterization:* The surface morphology and elemental distribution of Mg-Ti composites were examined using SEM equipped with EDS. Phase composition was analyzed by XRD using Co-Kα radiation over a 2θ range of 25°–85°.

*Compression Test:* Uniaxial compression tests were performed at room temperature on cylindrical specimens (Φ3 mm × 5 mm) using a universal testing machine (Instron, USA). Ti scaffolds with different pore sizes (*n* = 6 per group) and Mg-Ti composites (*n* = 6 per group) were evaluated. Bone tissue blocks (*n* = 4 per group) extending 2 mm above and below the implant were harvested from rat femurs at 4 weeks and 8 weeks post-implantation and subjected to compression testing under identical conditions. At least three samples were tested per group.

*Immersion Test: In vitro* immersion was conducted in SBF at 37 °C with daily solution changes with a solution volume-to-sample surface area ratio set to 20 mL/cm^2^ [46]. Mg-Ti discs (Φ10 mm × 5 mm) were ground, cleaned, and dried before testing. pH variation, hydrogen evolution, weight loss, and Mg ions release was measured. Mg ions concentration was determined using ICP-OES (Agilent, USA). Each test was performed in triplicate.

*Corrosion fatigue test:* Compression-compression fatigue tests were conducted on Φ4 mm × 9 mm Mg-Ti cylindrical specimens using a CARE M−6000 system. The tests were performed at a frequency of 10 Hz with a stress ratio of R = 0.1 (ratio of the absolute minimum to the maximum stress (4 MPa–40 MPa) in the cycle), under a simulated fluid flow rate of 120 mL/min [47]. Each test continued until specimen failure or 10^6^ loading cycles, at which point the run was terminated [48]. Fracture morphologies were examined by SEM. The debonding of the Mg-Ti composites was determined by analyzing the cross-sectional images acquired through SEM.

*Isolation and Culture of Primary Mouse BMSCs:* Femurs and tibias from 4-6-week-old male C57BL/6 mice were harvested under sterile conditions. Bone marrow was flushed with complete α-MEM (Gibco, USA) and centrifuged. Cells were resuspended and cultured at 37 °C with 5 % CO_2_. Non-adherent cells were removed after 48 h. BMSCs at passage 3 were used for experiments.

*Cytotoxicity Evaluation:* Ti and Mg-Ti samples (Φ10 mm × 2 mm) were extracted in culture medium at 37 °C for 72 h. The extracts were diluted to 16.7 % and used to culture BMSCs [49,50]. Cell morphology was assessed by phalloidin (Yeasen, China)/DAPI staining (Servicebio, China) and visualized with confocal microscopy. Cell viability was evaluated using CCK-8 assay at days 1, 3, and 5 (*n* = 6). For apoptosis analysis, BMSCs were cultured in extract for 5 days, stained with Annexin V/PI (Solarbio, China), and analyzed by flow cytometry.

*ALP and Alizarin Red S Staining:* To assess osteogenic differentiation, BMSCs were cultured in material extracts and stained for alkaline phosphatase (ALP) activity after 7 days using a commercial kit (Beyotime, China). Mineralization was evaluated by Alizarin Red S staining (Sigma-Aldrich, USA) after 21 days. Stained cells were imaged under a light microscope.

*RNA Extraction and qRT-PCR:* Gene expression related to osteogenesis (*Alp*, *Runx2*, *Opn*, *Ocn*) and osteoclastogenesis (*Ocstamp*, *Dcstamp*, *Ctsk*, *Pdgf-bb*, *Mmp9*) (Takara, Japan) was analyzed by qRT-PCR (*n* = 6). Total RNA was extracted from BMSCs and BMMs after induction, followed by reverse transcription. qRT-PCR was performed using *Gapdh* as a reference gene.

*Western Blot:* Protein expression of osteogenesis markers (RUNX2, OCN, OPN) in BMSCs and osteoclast-related markers (PLC-γ2, p-PLC-γ2, CTSK, Calcineurin, NFATc1) (Cohesion, China) in BMMs was evaluated by Western blot. Lamin-B1, β-tubulin, and GAPDH served as loading controls. Total protein was extracted, quantified, and subjected to SDS-PAGE, followed by PVDF membrane transfer, antibody incubation, and ECL detection.

*Enzyme-linked immunosorbent assay (ELISA):* PDGF-BB levels in culture supernatants were measured using a commercial ELISA kit (R&D Systems, USA) according to the manufacturer's instructions. Absorbance was read at 450 nm, and concentrations were calculated from a standard curve (*n* = 6).

*RNA Sequencing:* Total RNA from Control and Mg-Ti-1200-treated cells (osteogenic and osteoclastic induction) was extracted using Trizol and submitted for high-throughput sequencing. Library construction and sequencing were performed on the Illumina NovaSeq 6000 platform. Differential expression and functional enrichment analyses were conducted on the resulting data.

*TRAP Staining:* BMMs were cultured in 24-well plates for 5 days under indicated treatments. Cells were fixed and stained with a commercial TRAP kit (Hangzhou Yangming Biotechnology Co., Ltd). TRAP-positive multinucleated cells were counted in five random fields per well using light microscopy.

*F-Actin Ring Formation Assay:* BMSCs derived from 4-week-old mice were cultured with M-CSF (50 ng/mL) for 1 day, and then treated with M-CSF (30 ng/mL) and RANKL (100 ng/mL) for 4 days, either in the presence of the experimental materials or in their absence (Control). Cells were stained with phalloidin (Yeasen, China) and DAPI (Servicebio, China), and imaged using confocal microscopy (Olympus, Japan).

*Osteoclast Fusion Assay:* BMMs were induced with M-CSF and RANKL. After 3 days, cells were stained with Hoechst or CM-Dil (Beyotime, China), then co-cultured for 2 h. Fusion events were assessed under a fluorescence microscope and quantified using Image J.

*Bone Resorption Assay:* BMMs were seeded on bovine bone slices (Hangzhou Yangming Biotechnology Co., Ltd.) and cultured for 7 days. After removing cells, resorption pits were observed by SEM (Zesis, Germany). Resorbed areas were pseudocolored and quantified using Image J.

***T****ranswell Migration Assay:* HUVECs in serum-free α-MEM (1:1) were seeded into 8 μm Transwell inserts (Corning, USA), with complete medium in the lower chamber. After 24 h at 37 °C, inserts were fixed with 4 % paraformaldehyde, non-migrated cells removed, and migrated cells stained with 0.1 % crystal violet (Beyotime, China). Five random fields were imaged under a light microscope for cell counting.

*Tube Formation Assay:* Matrigel (Beyotime, China) was added to 48-well plates and polymerized at 37 °C. HUVECs were seeded and cultured in conditioned medium for 6 h. Tube structures were imaged (Nikon, Japan) and analyzed using Image J.

*Scratch Wound Assay:* HUVECs were seeded in 48-well plates and grown to confluence. A scratch was made using a 1 mL pipette tip, followed by incubation with conditioned medium mixed with complete α-MEM. Images were taken using an inverted fluorescence microscope (Nikon, Japan), and wound closure was quantified using Image J.

*Flow Cytometry:* BMSCs were induced osteogenically with different materials for 5 days, then stained using a FITC-Annexin V/PI apoptosis kit and analyzed by flow cytometry (BD Biosciences, USA). For osteoclast analysis, BMMs were isolated from 4-6-week-old C57BL/6 mice, cultured for 24 h, and red blood cells were lysed. After 5 days of induction, cells were stained with CD51 (APC), RANK (PE), CD11b (FITC) (ThermoFisher, USA), and PI, and analyzed by flow cytometry. For calcium analysis, cells were stained with Fluo-4 (Shandong Sparkjade Biotechnology Co., Ltd.) on days 1–5. CD11b^+^ CD51^−^ RANK^+^ cells were defined as precursors; CD11b^+^ CD51^+^ RANK^+^ cells as mature osteoclasts.

*Immunofluorescence:* Cells were fixed with 4 % paraformaldehyde, permeabilized with 0.25 % Triton X-100 (Beyotime, China), and blocked with 5 % BSA (Servicebio, China). Samples were incubated overnight at 4 °C with primary antibodies, followed by phalloidin (TRITC) or FITC-488-conjugated secondary antibodies (Cohesion, China) for 1 h. Nuclei were counterstained with DAPI. Images were acquired using a confocal microscope. For histology, bone tissues were fixed in 4 % paraformaldehyde and decalcified in 10 % EDTA. Paraffin sections (5 μm) were permeabilized, blocked, and stained with primary and secondary antibodies. Nuclei were stained with DAPI, and images were captured by confocal microscopy. Fluorescence intensity was analyzed using Image J in five random fields per sample.

*Femoral Defect Model and in vivo Evaluation:* Male SD rats (8 weeks, 200–300 g) were used under protocols approved by China Medical University (Project Number: CMU20242285). A 5 mm femoral defect was created using a 3 mm trephine, and biomaterials were implanted. Calcein Green and Alizarin Red were injected at weeks 8 to label bone formation. Femora and major organs were collected post-sacrifice. Fixed femora were scanned using micro-CT (μ50, SCANCO, Switzerland) for 3D reconstruction. Specimens were embedded in resin, sectioned (200 μm), ground (50 μm), and stained with Masson's trichrome to assess osteogenesis. The average degradation rate of the Mg phase was calculated in the three-dimensionally reconstructed material and surrounding newly formed bone using the degradation volume measured over the sampling time intervals. The rate was expressed as a percentage per day (%/day) according to the following formula:

(1) $$r=\frac{{\Delta V}_{Mg}}{V_{0}}\times \frac{100}{\Delta t}$$

where r represents the average degradation rate (%/day), ${\Delta V}_{Mg}$ is the degradation volume of the Mg phase, $V_{0}$ is the initial volume of the Mg phase, and Δt is the time interval (days).

*Finite Element Analysis:* Rat femoral imaging data were acquired from micro-CT images and imported into Mimics Research 19.0 software. Threshold segmentation, region growing, and three-dimensional model calculation were performed to establish three-dimensional models of the femur, porous scaffold, and newly formed bone. To simplify the computations, the newly formed bone was fused with the porous scaffold. The models were then imported into 3-matic software, where surface smoothing was applied, followed by the generation of surface meshes and volume meshes. The meshed models were exported in INP format and imported into Abaqus 2021 for finite element analysis. The proximal end of the femur was fixed in all six degrees of freedom, and a 20 N load was applied to the distal end (femoral condyle) to simulate the natural weight-bearing state of the rat.

*Statistical Analysis:* All data were presented as mean ± standard deviation (SD). Data was analyzed using Image J and GraphPad Prism 10.0 software. *p* < 0.05 is considered statistically significant, ∗*p* < 0.05, ∗∗*p* < 0.01, ∗∗∗*p* < 0.001, *p* ≥ 0.05 was considered as non-significant difference (ns).

## CRediT authorship contribution statement

**Wanxin Zheng:** Writing – review & editing, Writing – original draft, Data curation. **Sirui Tian:** Writing – review & editing, Writing – original draft, Investigation. **Jiaxing Huo:** Visualization. **Qiyue Zhang:** Validation. **Danning Wang:** Project administration, Formal analysis. **Zengqian Liu:** Supervision, Funding acquisition. **Baohong Zhao:** Resources, Funding acquisition. **Yuzhong Gao:** Supervision. **Zhefeng Zhang:** Supervision, Project administration. **Qiang Wang:** Supervision, Funding acquisition, Conceptualization.

## Data availability statement

The data that support the findings of this study are available from the corresponding author upon reasonable request.

## Ethics approval and consent to participate

The *in vivo* implantation experiment followed the Guide for the Care and Use of Laboratory Animals issued by the Ministry of Science and Technology of China and was approved by the Ethics Committee at China Medical University (approval number: CMU20251305/CMU20242285).

## Declaration of competing interest

The authors declare no conflict of interest.

## Abbreviation

**Mg:** Magnesium; **MSCs:** Mesenchymal Stem Cells; **Ti:** Titanium; **PDGF-BB:** Platelet-derived Growth Factor-BB; **ScRNA-seq:** Single-cell RNA sequencing; **NK cells:** Natural killers; **DCs:** Dendritic Cells; **ECs:** Endothelial Cells; **HSCs:** Hematopoietic Stem Cells; **GMPs:** Granulocyte-monocyte progenitors; **t-SNE:** t-distributed stochastic neighbor embedding; **OCPs:** Osteoclast Precursors; **FA-OCPs:** Fusion-activated OCPs; **MOCs:** Mature Osteoclasts; **SEM:** Scanning Electron Microscopy; **EDS:** Energy-dispersive X-ray Spectroscopy; **BMMs:** Bone Marrow Monocytes/Macrophages; **M-CSF:** Macrophage Colony-Stimulating Factor; **RANKL:** Receptor Activator of Nuclear Factor Kappa-B Ligand; **ELISA:** Enzyme-Linked Immunosorbent Assay; **BS/TS:** Bone surface area to Total surface ratio; **BV/TV:** Bone Volume area to Total volume ratio; **XRD:** X-Ray diffraction; **CCK-8:** Cell counting kit-8; **KEGG:** Kyoto Encyclopedia of Genes and Genomes; **Tb.N:** Trabecular number; **Tb.Th:** Trabeculae thickness; **FEA:** Finite element analysis**.**

## References

[1] Zhao D., Yu K., Sun T. (2023). Material–structure–function integrated additive manufacturing of degradable metallic bone implants for load-bearing applications. Adv. Funct. Mater..

[2] Li J., Ebied M., Xu J. (2018). Current approaches to bone tissue engineering: the interface between biology and engineering. Adv. Healthcare Mater..

[3] Hu B., Li Y., Wang M. (2018). Functional reconstruction of critical-sized load-bearing bone defects using a Sclerostin-targeting miR-210-3p-based construct to enhance osteogenic activity. Acta Biomater..

[4] Zhang L., Yang G., Johnson B. (2019). Three-dimensional (3D) printed scaffold and material selection for bone repair. Acta Biomater..

[5] Yuan B., Liu P., Zhao R. (2023). Functionalized 3D-printed porous titanium scaffold induces *in situ* vascularized bone regeneration by orchestrating bone microenvironment. J. Mater. Sci. Technol..

[6] Ahn T., Lee D., Kim T. (2018). Modification of titanium implant and titanium dioxide for bone tissue engineering. Adv. Exp. Med. Biol..

[7] Cai Z., Du P., Li K. (2024). A review of the development of titanium-based and magnesium-based metallic glasses in the field of biomedical materials. Materials.

[8] Sarkar N., Susmita B. (2020). Controlled delivery of curcumin and vitamin K2 from hydroxyapatite-coated titanium implant for enhanced *in vitro* chemoprevention, osteogenesis, and *in vivo* osseointegration. ACS Appl. Mater. Interfaces.

[9] Benady A., Meyer S., Golden E. (2023). Patient-specific Ti-6Al-4V lattice implants for critical-sized load-bearing bone defects reconstruction. Mater. Des..

[10] Abd-Elaziem W., Darwish M., Hamada A. (2024). Titanium-based alloys and composites for orthopedic implants applications: a comprehensive review. Mater. Des..

[11] Prasad K., Bazaka O., Chua M. (2017). Metallic biomaterials: current challenges and opportunities. Materials.

[12] Huang X., Lou Y., Duan Y. (2024). Biomaterial scaffolds in maxillofacial bone tissue engineering: a review of recent advances. Bioact. Mater..

[13] Duda G., Geissler S., Checa S. (2023). The decisive early phase of bone regeneration. Nat. Rev. Rheumatol..

[14] Zhou H., Liang B., Jiang H. (2021). Magnesium-based biomaterials as emerging agents for bone repair and regeneration: from mechanism to application. J. Magnesium Alloys.

[15] Salhotra A., Shah H., Levi B. (2020). Mechanisms of bone development and repair. Nat. Rev. Mol. Cell Biol..

[16] Winkler T., Sass F., Duda G. (2018). A review of biomaterials in bone defect healing, remaining shortcomings and future opportunities for bone tissue engineering. Bone Joint Res..

[17] Zhang Y., Yu T., Xiang Q. (2025). Osteoclasts drive bone formation in ectopic and orthotopic environments. Biomaterials.

[18] Sims N., Martin T. (2020). Osteoclasts provide coupling signals to osteoblast lineage cells through multiple mechanisms. Annu. Rev. Physiol..

[19] Xie H., Cui Z., Wang L. (2014). PDGF-BB secreted by preosteoclasts induces angiogenesis during coupling with osteogenesis. Nat. Med..

[20] Quan H., Ren C., Xie H. (2025). An injectable hydrogel loaded with miRNA nanocarriers promotes vessel-associated osteoclast (VAO)-mediated angiogenesis and bone regeneration in osteonecrosis of the rat femoral head. Biomaterials.

[21] Zhen G., Dan Y., Wang R. (2021). An antibody against Siglec-15 promotes bone formation and fracture healing by increasing TRAP^+^ mononuclear cells and PDGF-BB secretion. Bone Res..

[22] Chen Z., Yang W., Tang Y. (2025). Trifunctional sialylation-based SF-ZIF@NA hydrogel for selective osteoclast inhibition and enhanced bone-vessel regeneration in osteoporotic bone defects. Adv. Sci..

[23] Liu W., Wang Q., Luo H. (2024). Nanographene oxide promotes angiogenesis by regulating osteoclast differentiation and platelet-derived growth factor secretion. ACS Nano.

[24] Wang J., Xu J., Hopkins C. (2020). Biodegradable magnesium-based implants in orthopedics-A general review and perspectives. Adv. Sci..

[25] Zheng N., Xu J., Ruan Y. (2022). Magnesium facilitates the healing of atypical femoral fractures: a single-cell transcriptomic study. Mater. Today.

[26] Wang N., Yang S., Shi H. (2022). Magnesium alloys for orthopedic applications: a review on the mechanisms driving bone healing. J. Magnesium Alloys.

[27] Yang Y., He C., E D. (2020). Mg bone implant: features, developments and perspectives. Mater. Des..

[28] Chen S., Liu F., Xin H. (2024). Boosting MRSA infectious osteoporosis treatment: Mg-doped nanofilm on vacancy-enriched TiO_2_ coating for providing *in situ* sonodynamic bacteria-killing and osteogenic alkaline microenvironment. Adv. Funct. Mater..

[29] Hassan N., Krieg T., Kopp A. (2024). Challenges and pitfalls of research designs involving magnesium-based biomaterials: an overview. Int. J. Mol. Sci..

[30] Akbarzadeh F., Sarraf M., Ghomi E. (2024). A state-of-the-art review on recent advances in the fabrication and characteristics of magnesium-based alloys in biomedical applications. J. Magnesium Alloys.

[31] Li J., Zhao C., Xu Y. (2022). Remodeling of the osteoimmune microenvironment after biomaterials implantation in murine tibia: Single-cell transcriptome analysis. Bioact. Mater..

[32] Vesprey A., Suh E., Aytürk D Göz (2021). Tmem100- and Acta2-Lineage cells contribute to implant isseointegration in a mouse model. J. Bone Miner. Res..

[33] Ben Amara H., Martinez D., Shah F. (2023). Magnesium implant degradation provides immunomodulatory and proangiogenic effects and attenuates peri-implant fibrosis in soft tissues. Bioact. Mater..

[34] Ben Amara H., Martinez D., Iskhakova K. (2025). Multifaceted bone response to immunomodulatory magnesium implants: osteopromotion at the interface and adipogenesis in the bone marrow. Biomaterials.

[35] Tsukasaki M., Huynh N., Okamoto K. (2020). Stepwise cell fate decision pathways during osteoclastogenesis at single-cell resolution. Nat. Metab..

[36] Dou C., Zhang M., Ren D. (2023). Bi-continuous Mg-Ti interpenetrating-phase composite as a partially degradable and bioactive implant material. J. Mater. Sci. Technol..

[37] Han X., Zhou L., Liu Z. (2024). Degradation behavior of biomedical partially degradable Ti–Mg composite fabricated by 3D printing and pressureless infiltration. J. Mater. Res. Technol..

[38] Sanchez A., Luthringer B., Feyerabend F. (2015). Mg and Mg alloys: how comparable are *in vitro* and *in vivo* corrosion rates? A review. Acta Biomater..

[39] Zhang B., Yang Y., Yi J. (2021). Hyperglycemia modulates M1/M2 macrophage polarization via reactive oxygen species overproduction in ligature-induced periodontitis. J. Periodontal. Res..

[40] Cao H., Shi K., Long J. (2025). PDGF-BB improves cortical bone quality through restoring the osteogenic microenvironment in the steroid-associated osteonecrosis of rabbits. J. Orthopaedic Translat..

[41] Kang J., Kang N., Yang Y. (2020). The role of Ca^2+^-NFATc1 signaling and its modulation on osteoclastogenesis. Int. J. Mol. Sci..

[42] Gou H., Wang T., Chen Y. (2025). Role of Pink1 in regulating osteoclast differentiation during periodontitis. J. Dent. Res..

[43] Ma Y., Di R., Zhao H. (2022). P2X7 receptor knockdown suppresses osteoclast differentiation by inhibiting autophagy and Ca^2+^/calcineurin signaling. Mol. Med. Rep..

[44] Karagiota A., Mylonis I., Simos G. (2019). Protein phosphatase PPP3CA (calcineurin A) down-regulates hypoxia-inducible factor transcriptional activity. Arch. Biochem. Biophys..

[45] Imai Y., Ohta E., Takeda S. (2016). Histone deacetylase inhibitor panobinostat induces calcineurin degradation in multiple myeloma. JCI Insight.

[46] ASTM-G31-72 (2004).

[47] Han L., Zhang Z., Dai J. (2023). *In vitro* bio-corrosion behaviors of biodegradable AZ31B magnesium alloy under static stresses of different forms and magnitudes. J. Magnesium Alloys.

[48] Liu J., Tang H., Tan L. (2025). Corrosion fatigue behavior and mechanism of Mg-Zn-Zr-Nd alloy in protein-containing simulated body fluid. Int. J. Fatig..

[49] ISO 10993-12 (2012).

[50] Wang J., Witte F., Xi T. (2015). Recommendation for modifying current cytotoxicity testing standards for biodegradable magnesium-based materials. Acta Biomater..

