# Supplementary material for: Mechanically adaptive Mg-Ti composites guided by single-cell insights accelerate load-bearing bone regeneration via dual modulation of osteogenesis and osteoclastogenesis
Source: Bioact Mater. 2025 Nov 6;57:54–72. doi: 10.1016/j.bioactmat.2025.10.038 (PMC12639329; doi:10.1016/j.bioactmat.2025.10.038)
Supplement: Multimedia component 1 [file mmc1.docx]

**Supporting Information**

**Mechanically Adaptive** **Mg-Ti Composites Guided by Single-Cell Insights**

**Accelerate Load‑Bearing Bone Regeneration**

**Via Dual Modulation of Osteogenesis and Osteoclastogenesis**

**Supplementary tables**

**Supplementary Table S1. Mg-Ti composites’ magnesium content and volume fraction.**

**
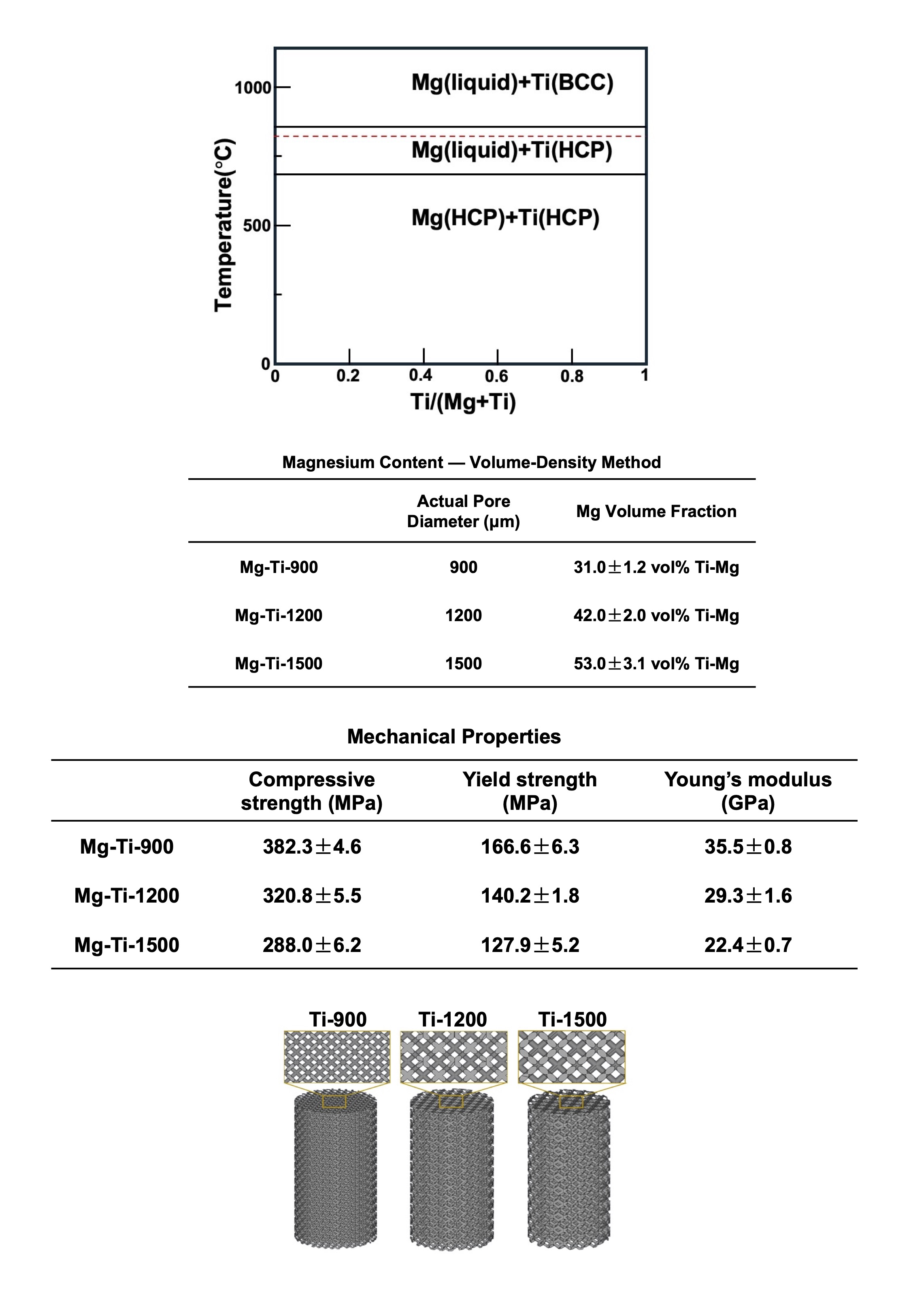
**

**Supplementary Table S2. Mg-Ti composites’ mechanical properties.**


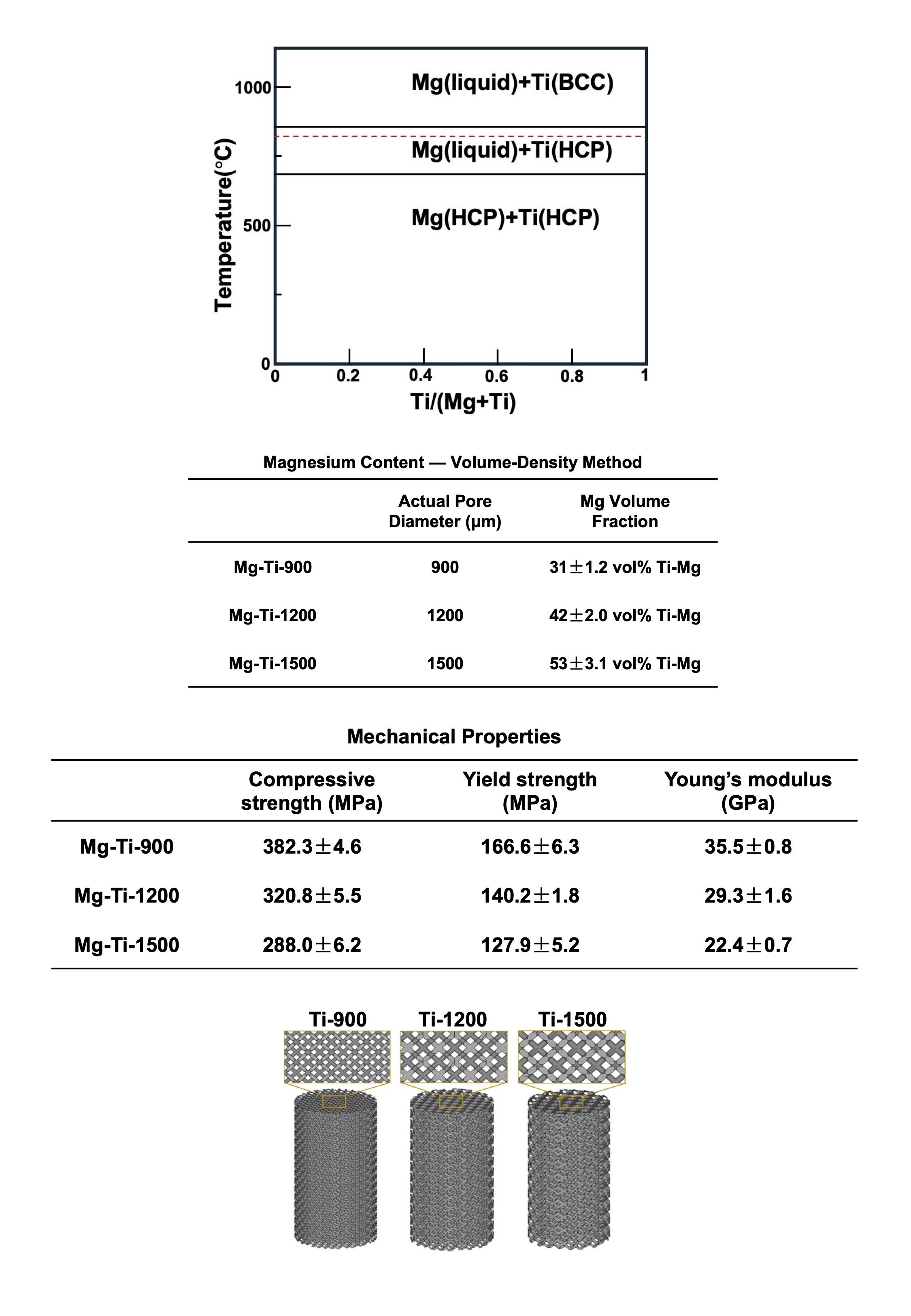


**Supplementary figures**


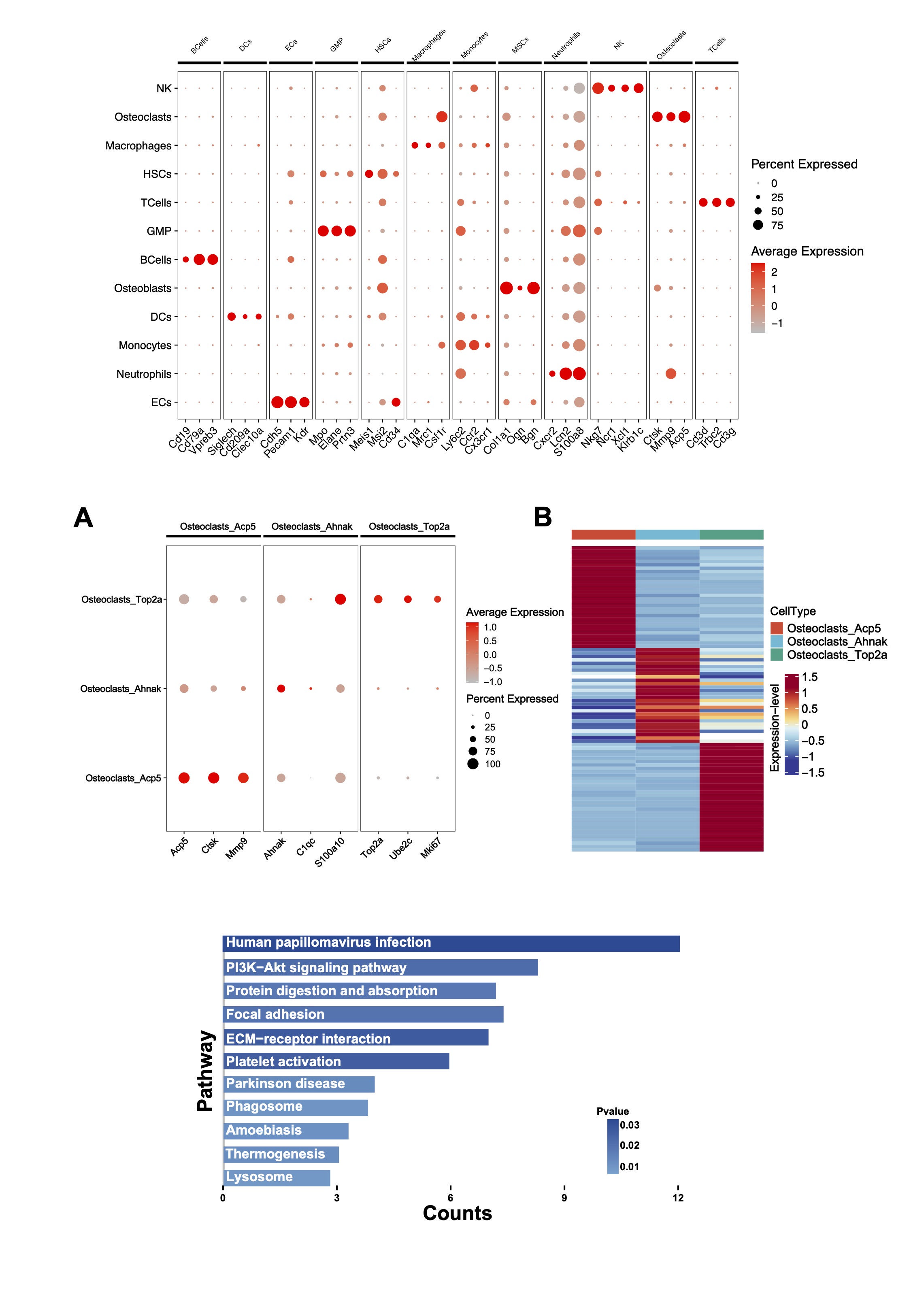


**Supplementary Figure S1. Marker gene expression across major cell types.**
Dot plot shows the expression of representative marker genes across major cell types identified by single-cell RNA sequencing. Dot size reflects the percentage of cells expressing each gene within a given cell type, and color intensity indicates the average expression level. Marker genes are grouped by biological function to highlight distinct transcriptional profiles of each cell population.


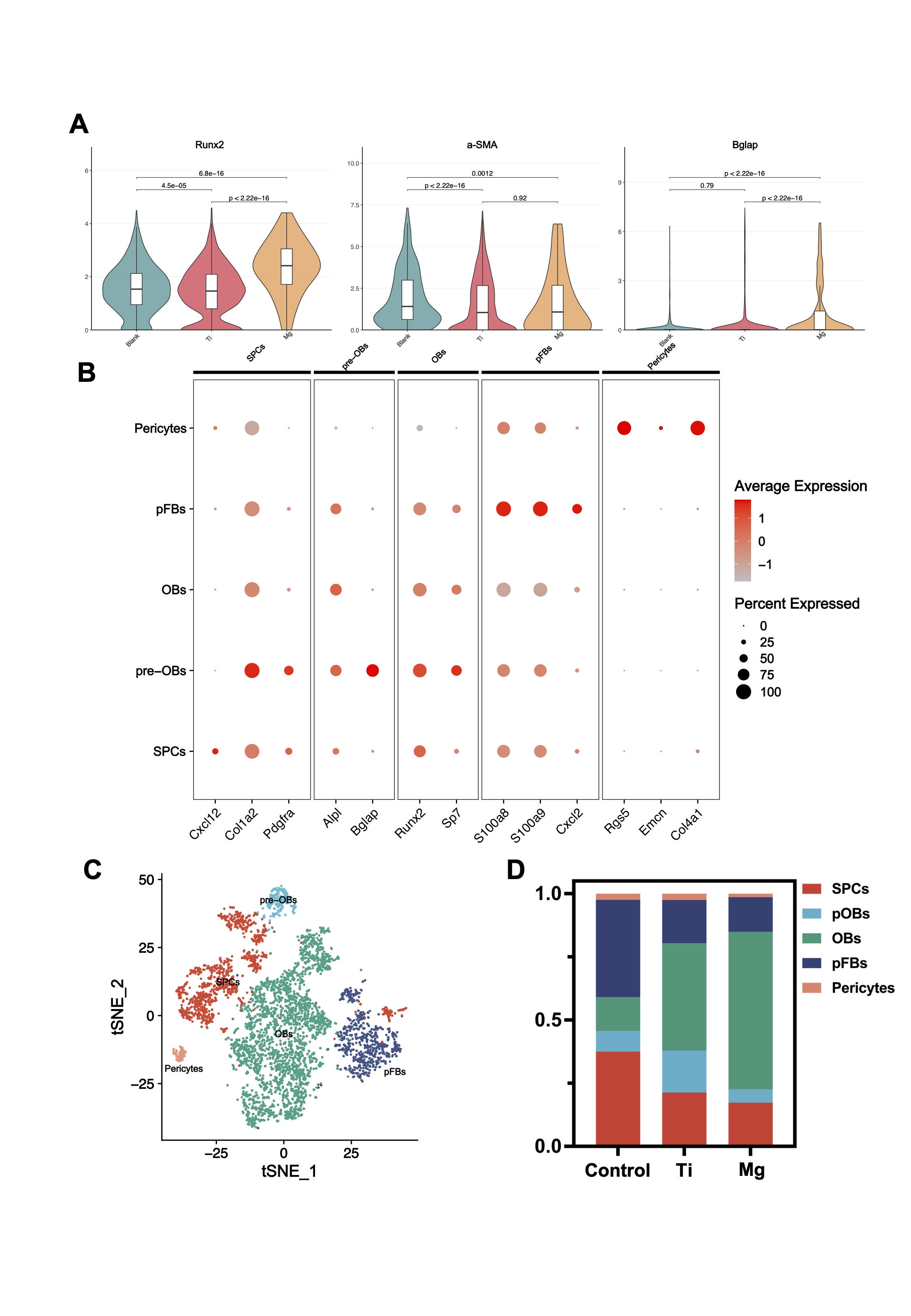


**Supplementary Figure S2. Heterogeneity analysis of mesenchymal stem cells (MSCs) populations.**

(A) Violin plots of relative expression for Runx2, α-SMA, and Bglap in MSCs, with p-values for group comparisons. (B) Dot plot of MSCs subpopulations Pericytes, Proliferative Fibroblasts (pFBs), Osteoblasts (OBs), Pre-osteoblasts (pre-OBs), Skeletal Progenitor Cells (SPCs) based on marker genes. (C) t-SNE plot showing MSCs subpopulation clusters: SPCs, pre-OBs, OBs, pFBs, Pericytes. (D) Stacked bar chart of subpopulation proportions (SPCs, pre-OBs, OBs, pFBs, Pericytes) under conditions (Control; Ti; Mg).


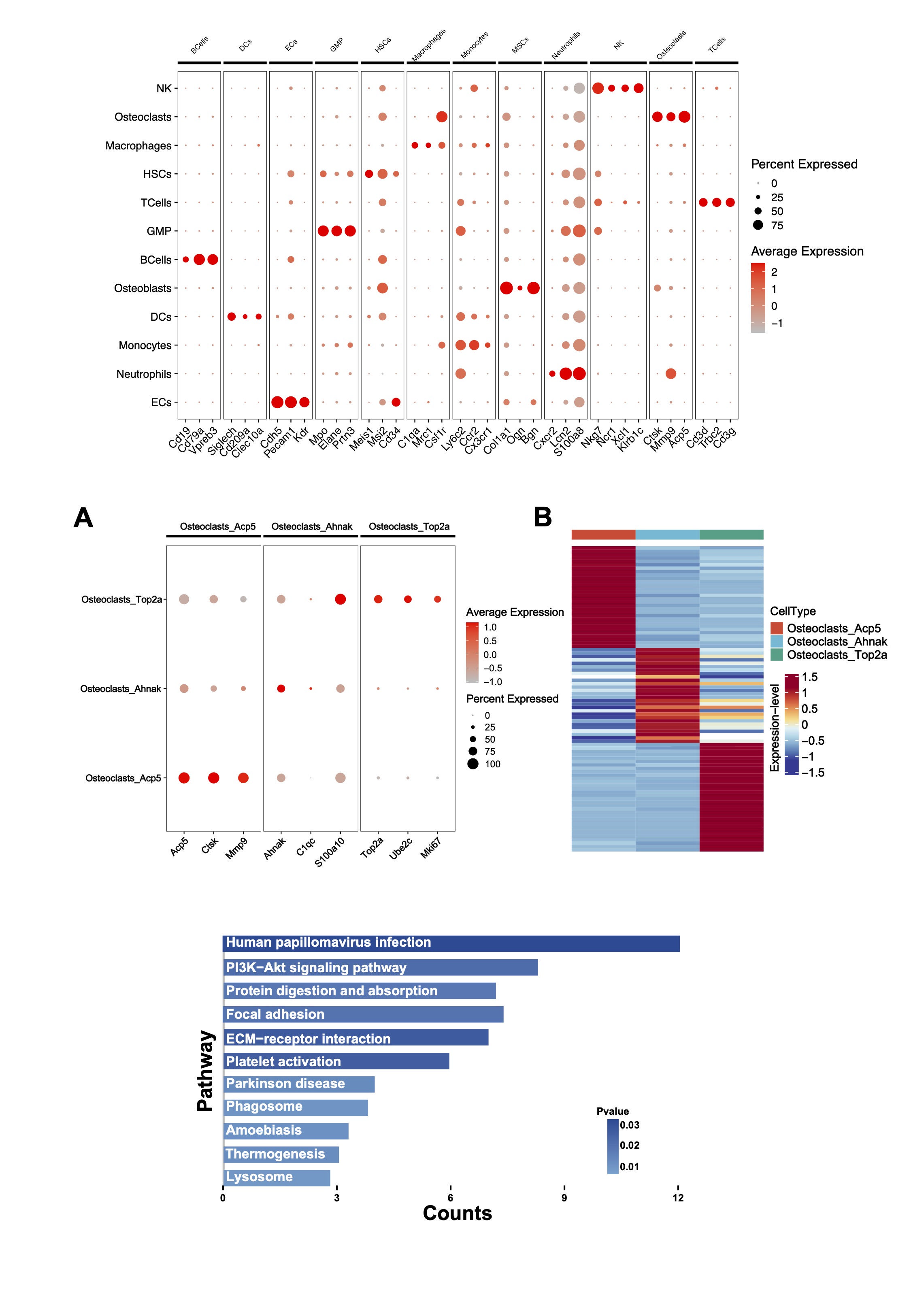


**Supplementary Figure S3. Expression of marker genes across osteoclast subtypes.**
(A) Dot plot shows the expression of key marker genes across three identified osteoclast subtypes: Osteoclasts-Acp5 (MOC), Osteoclasts-Ahnak (OCP), and Osteoclasts-Topo2a (FA-OCP). The size of each dot indicates the percentage of cells expressing the gene, and the color intensity represents the average expression level. (B) Heatmap of marker gene expression levels across the osteoclast subtypes. The expression values are normalized, with red indicating higher expression and blue indicating lower expression. The cell type classification is shown in the legend, with distinct profiles observed for each osteoclast subtype.


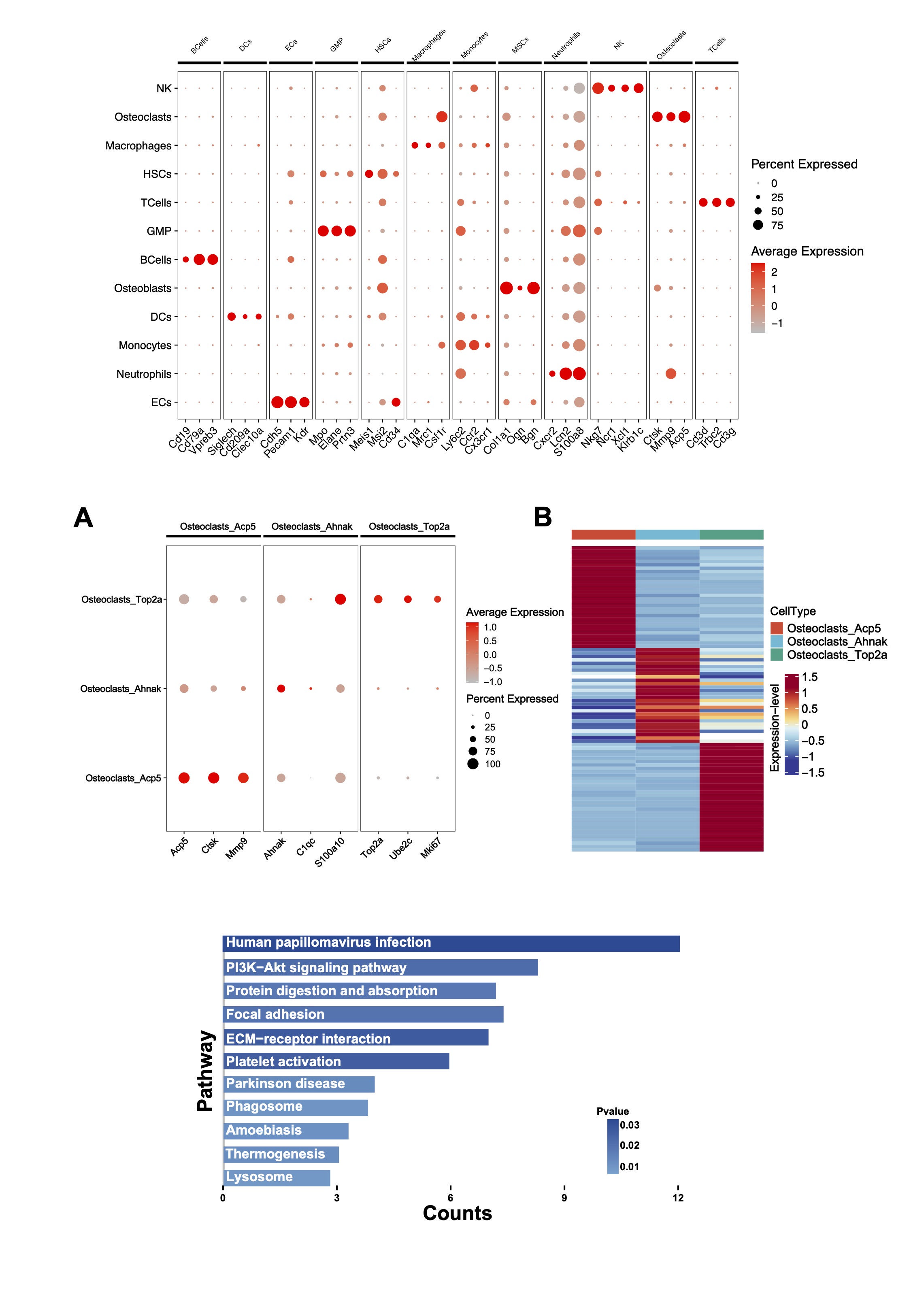


**Supplementary Figure S4. KEGG pathway enrichment analysis between Ti and Control groups.**
Bar plot displays the top differentially enriched KEGG pathways between the Ti and Control groups. The enrichment score is shown on the x-axis, and the color intensity reflects the degree of enrichment. Pathways are ranked based on statistical significance and enrichment strength.

**
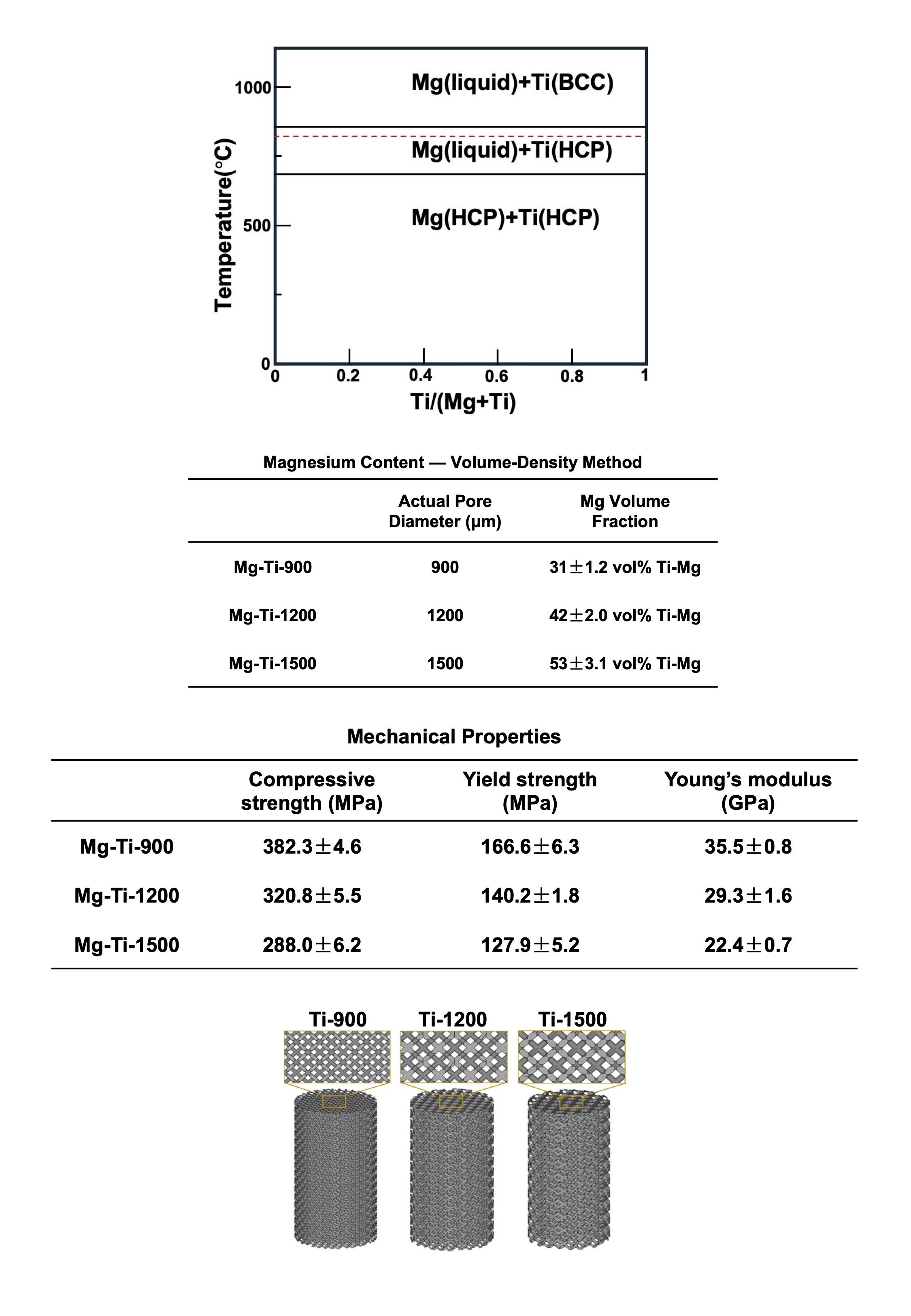
**

**Supplementary Figure S5. Visualization of titanium scaffolds with varying pore sizes.**
The three titanium scaffolds (Ti-900, Ti-1200, and Ti-1500) are shown, each featuring distinct pore architectures. The upper inset magnifications highlight the unique differences in pore structure at the microscopic level.


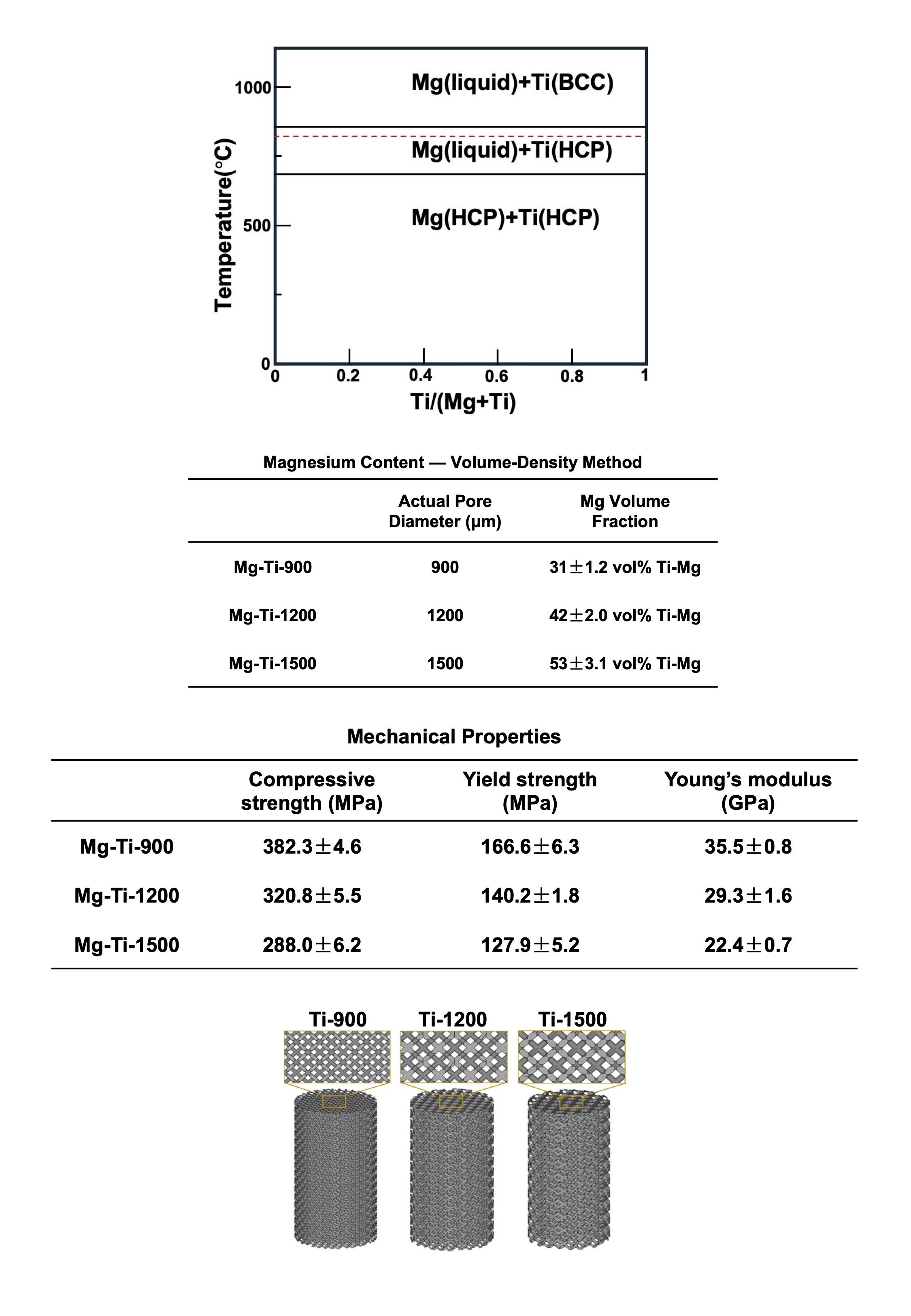


**Supplementary Figure S6. Selection of optimal temperature for the diffusion of magnesium into titanium scaffold.**
This phase diagram illustrates the temperature-dependent melting behavior of Mg and Ti phases at various compositions. The graph presents three distinct phases: **1. Mg (liquid) + Ti (BCC)**, **2. Mg (liquid) + Ti (HCP)**, **3. Mg (HCP) + Ti (HCP)**. The red dashed line at **850 °C** indicates the optimal temperature for the **pressureless infiltration** of Mg into the Ti scaffold, where both the liquid phase of Mg and the desired crystal structure of Ti (HCP) coexist.


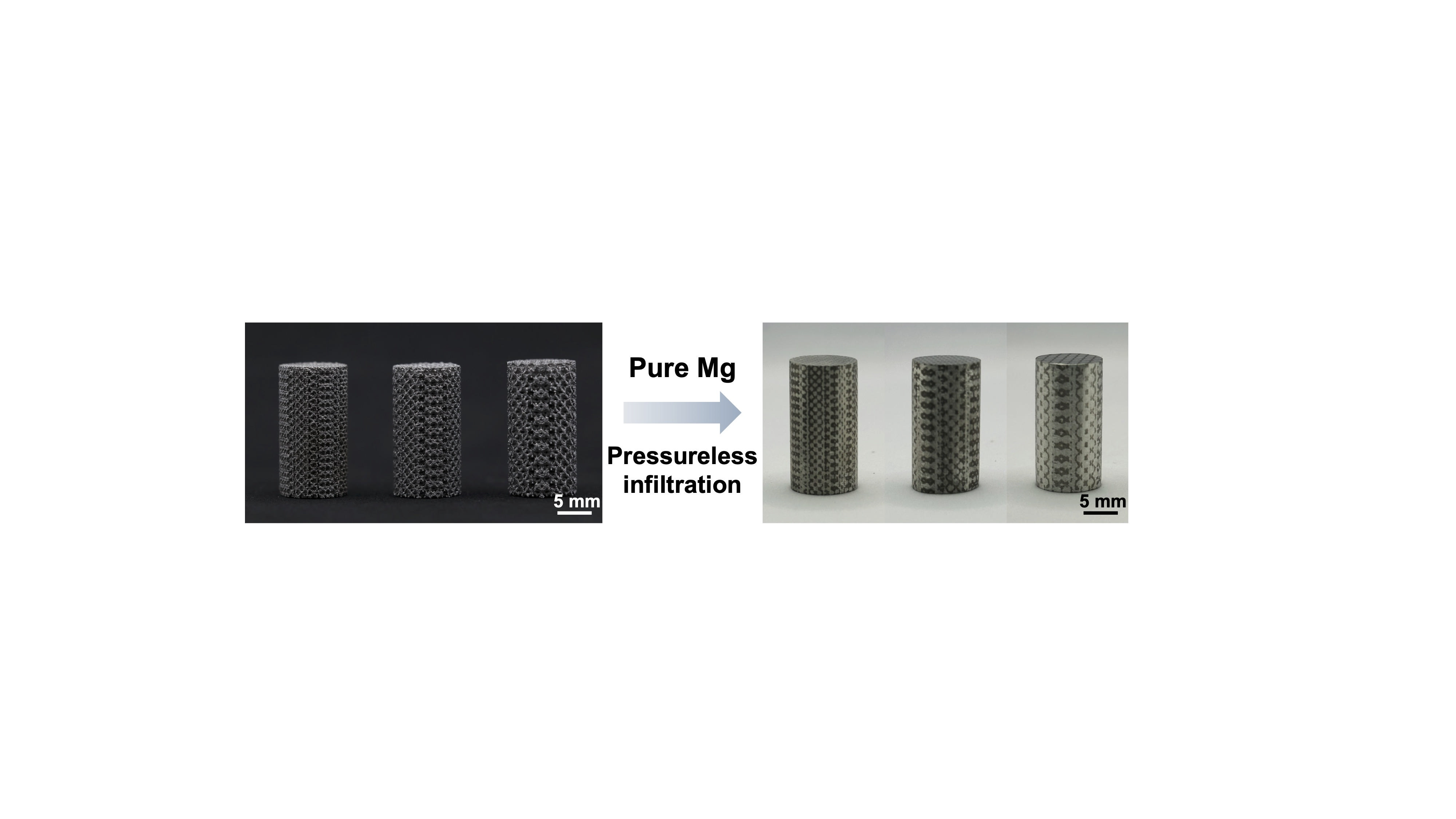


**Supplementary Figure S7. Pressureless infiltration process for Mg-Ti composites formation.**


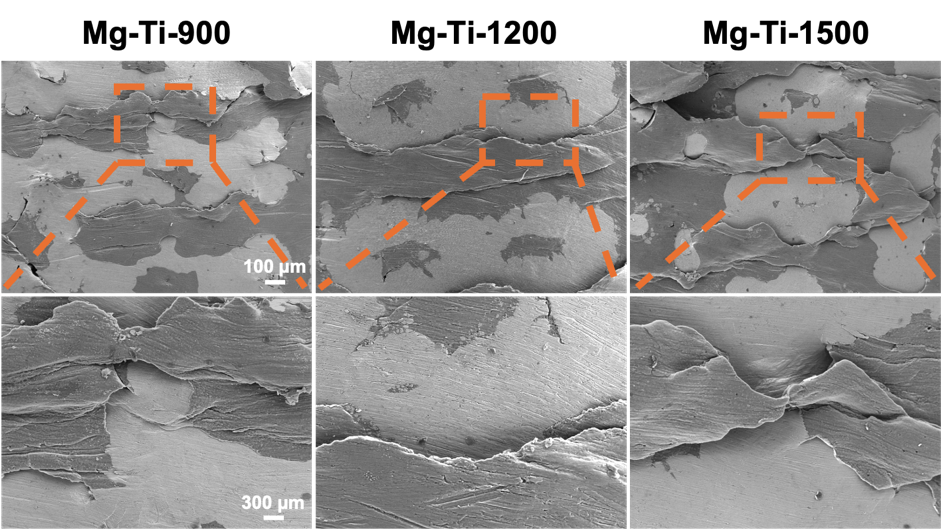


**Supplementary Figure S8. Surface morphology of compressed Mg-Ti composites.**
SEM images display the surface morphology of three different Mg-Ti composites after compression. The dashed orange lines highlight areas where **interface cracking** between the Mg and Ti phases is observed. Despite the presence of cracks, the interaction between the two phases effectively **suppresses the propagation of interface cracks**. The scale bars represent **100 μm** (top row) and **300 μm** (bottom row).


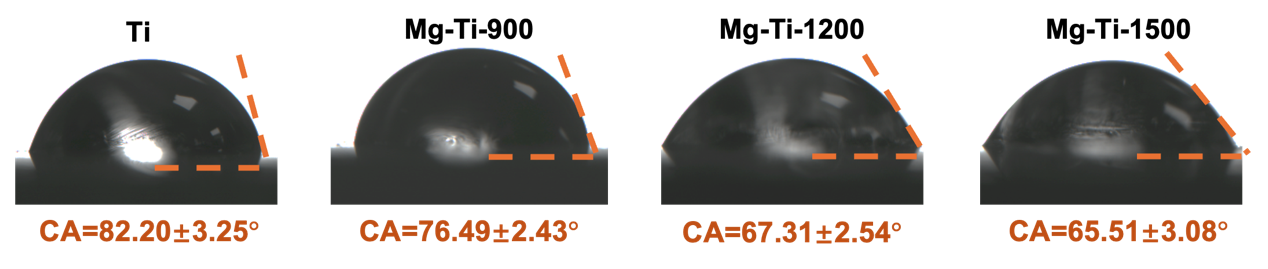


**Supplementary Figure S9. Contact angle measurements of materials with varying Mg content.**
The images show the contact angles (CA) of four different materials with varying amounts of Mg phase. As the Mg content increases, the contact angle decreases, indicating enhanced hydrophilicity. The values presented under each image represent the measured contact angle (CA) ± standard deviation. The results suggest that higher Mg content in the composites leads to a more hydrophilic surface, improving their wettability compared to materials with lower Mg fractions.


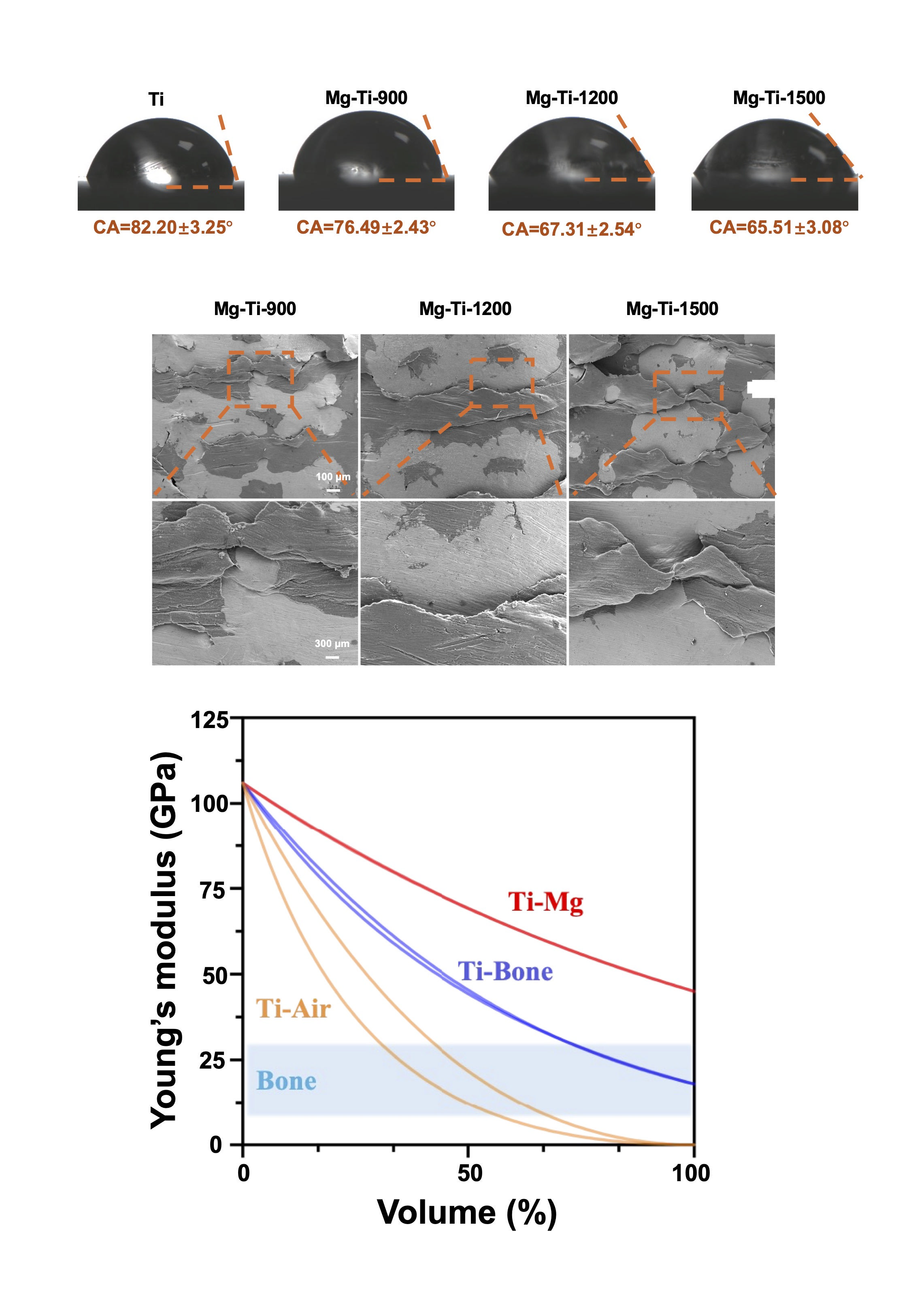


**Supplementary Figure S10. Elastic modulus of Mg-Ti composite under different phase volume fractions.**
This graph shows the elastic modulus of Mg-Ti composite as a function of the volume fractions of three different phases: Mg, bone, and air within a Ti scaffold. Each data point corresponds to a material configuration where one specific phase dominates the pore space, with the x-axis representing the volume fraction of that phase. The elastic modulus was estimated using Nelson’s law, demonstrating a linear relationship between phase composition and mechanical stiffness. The results highlight how varying the infilled phase modulates the scaffold’s elastic properties to match bone-like mechanics.

**
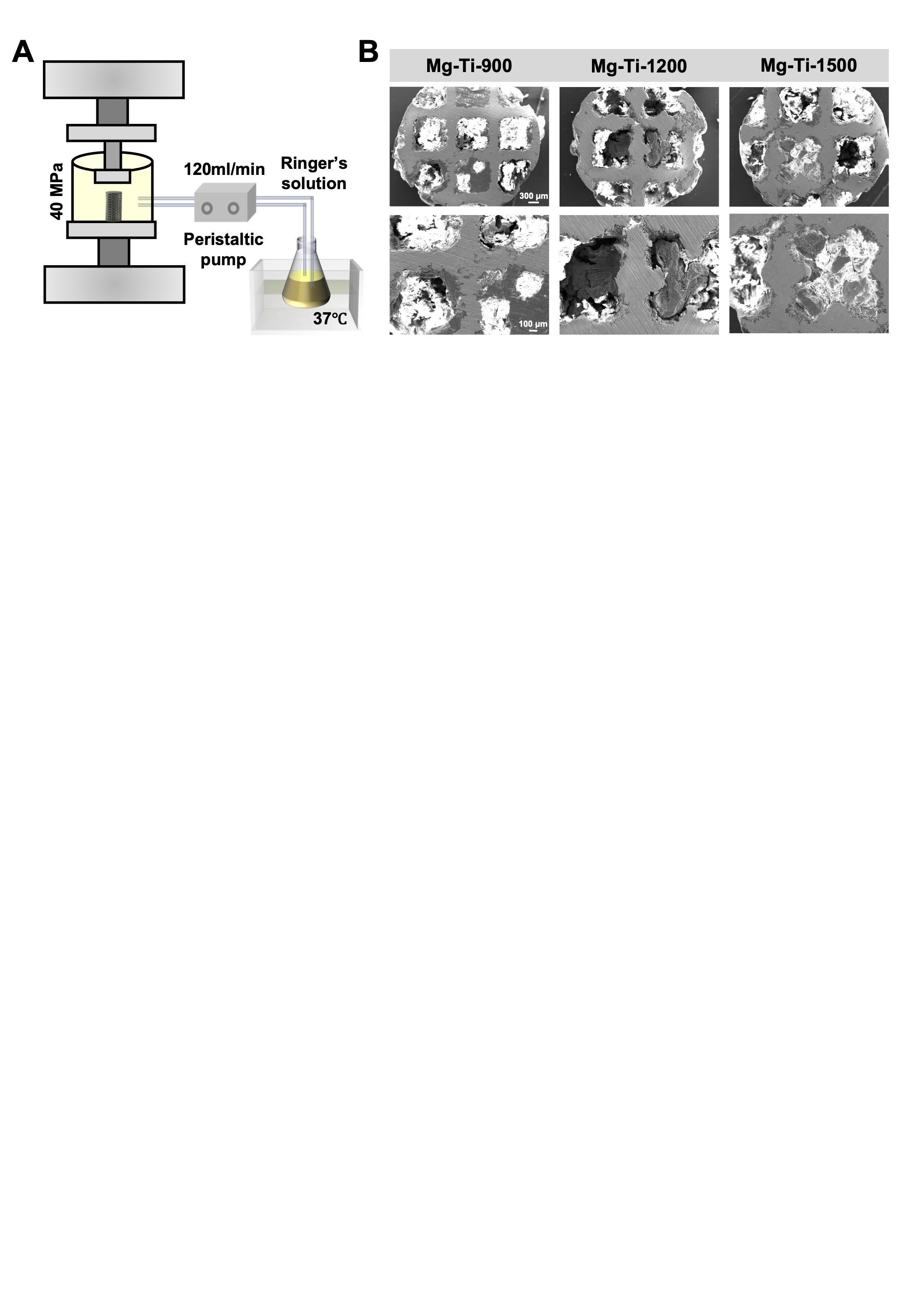
**

**Supplementary Figure S11. Corrosion fatigue test of Mg-Ti composites**

(A) Schematic illustration of the corrosion fatigue testing setup, featuring Ringer’s solution delivered at a flow rate of 120 mL/min via a peristaltic pump and maintained at 37 °C. (B) Cross-

sectional SEM images of Mg-Ti composites after corrosion fatigue testing. (Top row: 300 μm scale bar; bottom row: 100 μm scale bar)

**
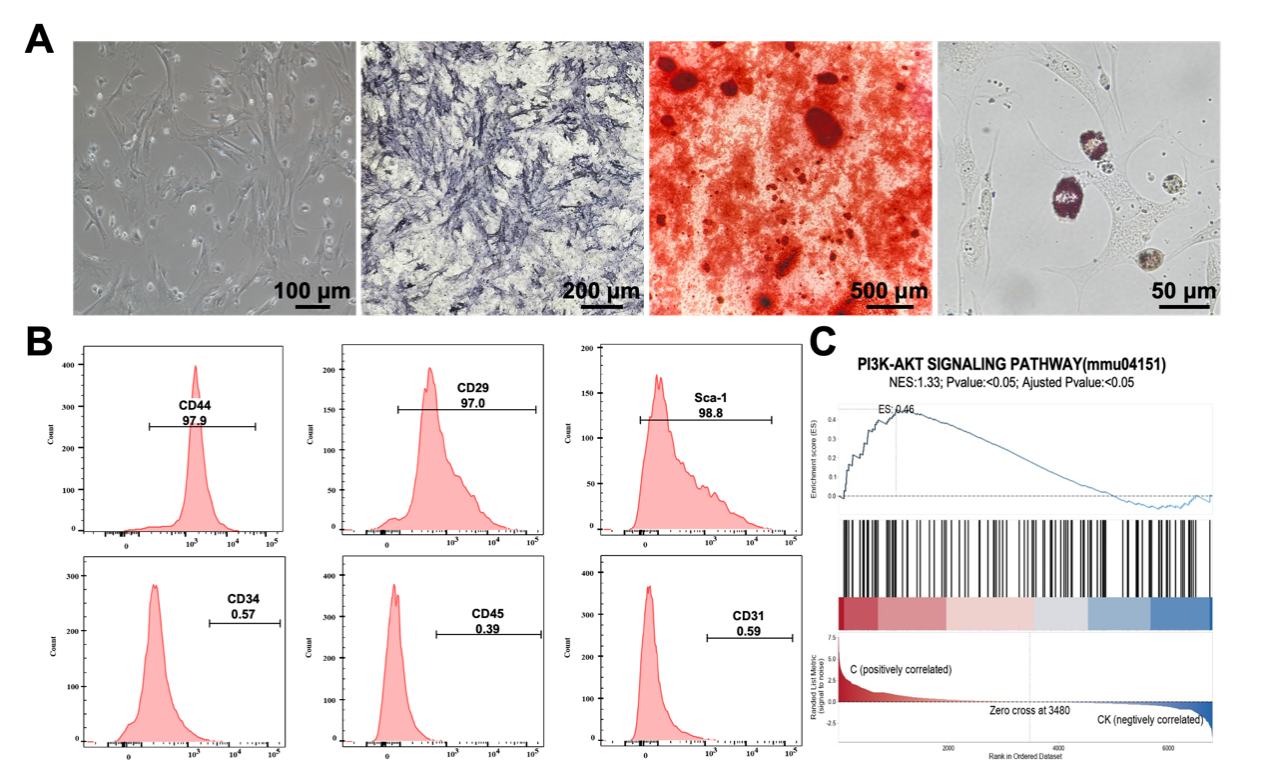
**

**Supplementary Figure S12. Characterization of mouse bone marrow mesenchymal stem cells (BMSCs)**
**(A)** Morphological images of BMSCs at various stages, including: 1. **Cellular morphology** under light microscopy (top left, scale bar = 100 µm). 2. **ALP staining** for osteogenic differentiation (top middle, scale bar = 200 µm). 3. **ARS staining** for mineralized nodule formation during osteogenesis (top right, scale bar = 500 µm). 4. **Oil red O staining** for adipogenic differentiation (bottom right, scale bar = 50 µm). **(B)** Flow cytometry analysis of six surface markers for cell identification and purity assessment. The expression of markers CD44 (97.9%), CD29 (97.0%), and Sca-1 (98.8%) confirm the successful isolation of mesenchymal stem cells with high purity, while CD34, CD45, and CD31 are low. **(C)** PI3K-Akt signaling pathway activation in osteogenic differentiation, with significant enhancement upon exposure to Mg-Ti-1200 material (right panel). The pathway analysis shows increased enrichment in the PI3K-Akt signaling pathway (NES = 1.33, p-value < 0.05, adjusted p-value < 0.05), suggesting that Mg-Ti-1200 promotes osteogenic differentiation via PI3K-Akt pathway activation.

**
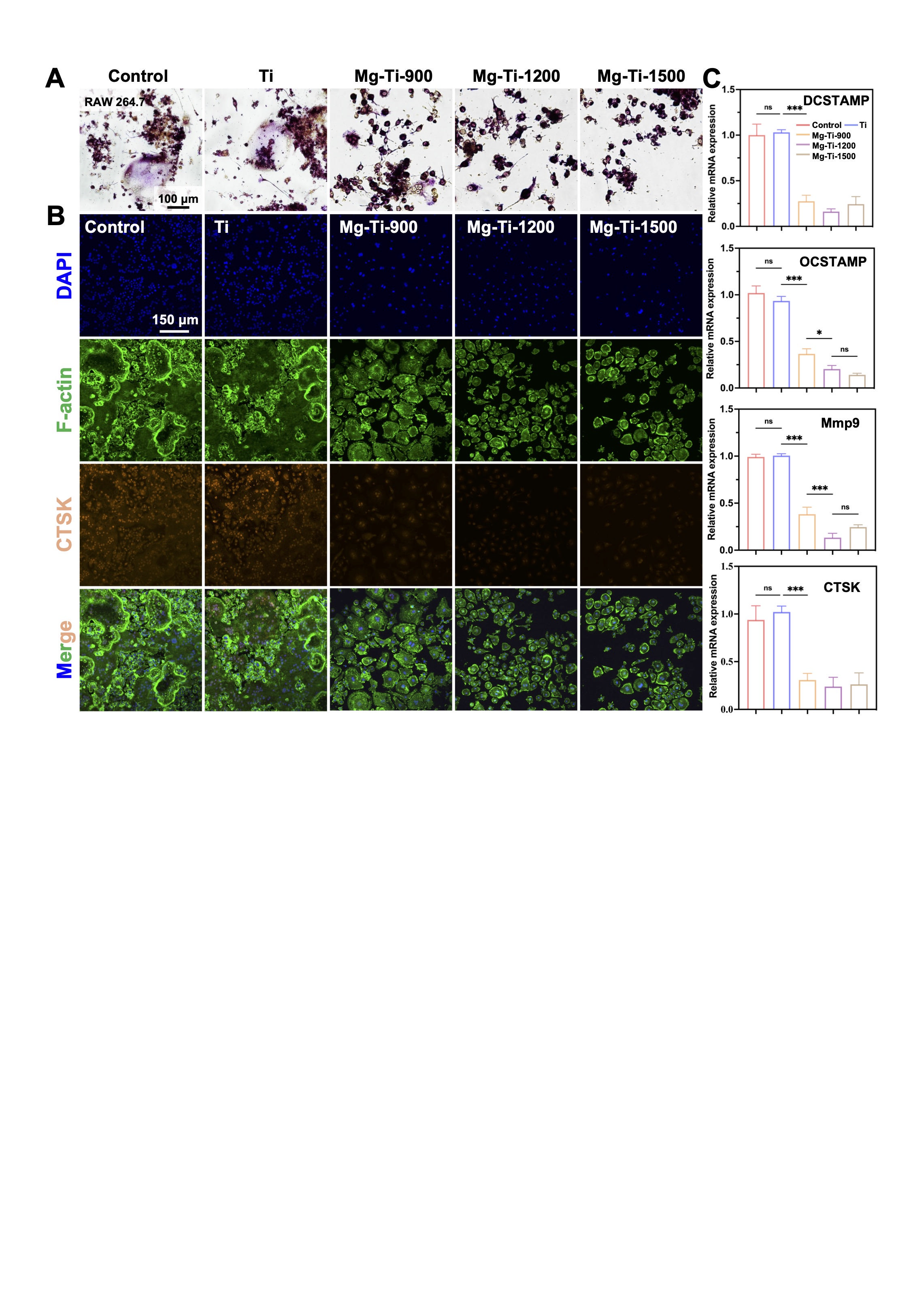
**

**Supplementary Figure S13. Osteoclast differentiation with different treatments.**
**(A)** TRAP staining of osteoclasts differentiated from RAW 264.7 cells after treatment with various materials. The images show the formation of TRAP-positive multinucleated cells with varying intensity across the treatment groups: **Control**, **Ti**, **Mg-Ti-900**, **Mg-Ti-1200**, and **Mg-Ti-1500** (Scale bar = 100 µm). **(B)** Immunofluorescence staining for CTSK (Cathepsin K), a marker of mature osteoclasts, in cells treated with different materials (Scale bar = 200 µm). **(C)** qRT-PCR validation of osteoclast-specific genes in cells treated with different materials. The data are shown as the mean ± SD (*n* = 6); *P < 0.05; **P < 0.01; ***P < 0.001 indicated significant differences between the indicated columns (one-way ANOVA).

**
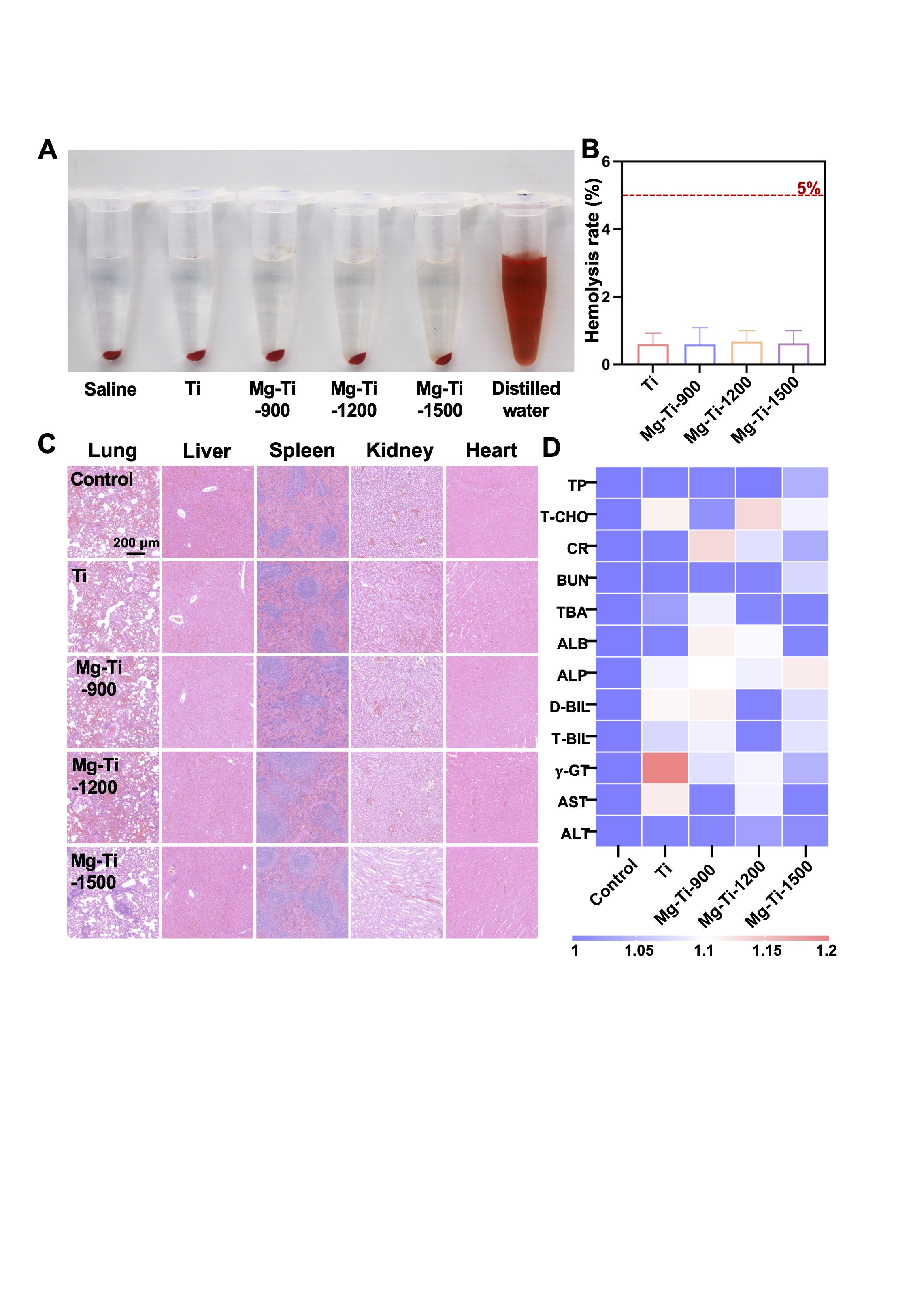
**

**Supplementary Figure S14. Biocompatibility and safety evaluation of implanted materials.**
**(A)** Hemolysis test images shows the lack of red blood cell lysis in the material groups, indicating that the materials are non-hemolytic. **(B)** Hemolysis rate for each treatment group (Control, Ti, Mg-Ti-900, Mg-Ti-1200, Mg-Ti-1500). The results show that all groups exhibit hemolysis rates significantly lower than the safety threshold of 5%. **(C)** Histological analysis of five major organs (liver, kidney, lung, heart, and spleen) after 8 weeks of material implantation (Scale bar = 200 µm). **(D)** Blood test results after 8 weeks of implantation.

**
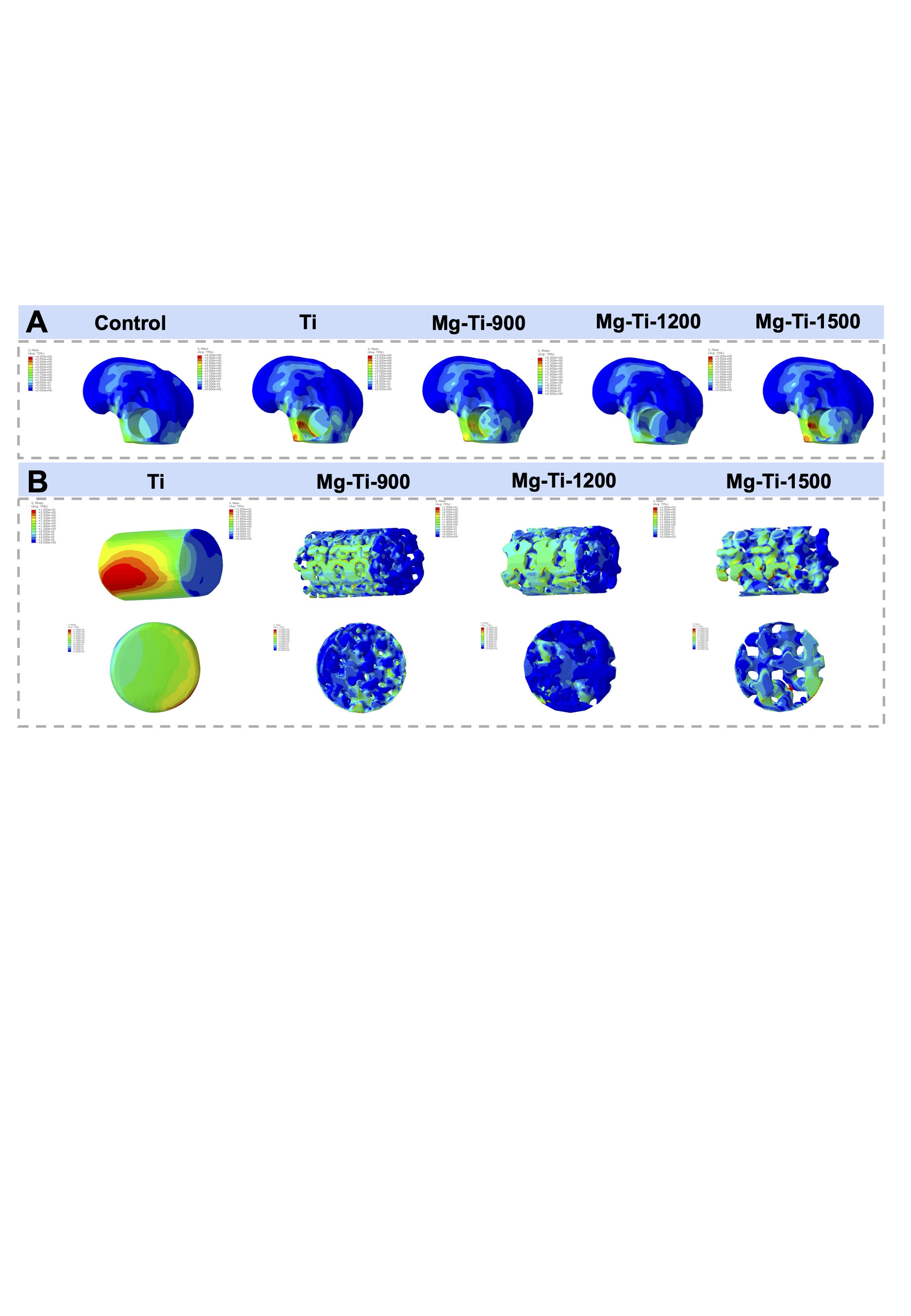
**

**Supplementary Figure S15. Finite element analysis (FEA) of stress distribution in rat femoral bone-implant complexes 8 weeks post-implantation.**

(A) FEA simulation depicts the stress on the surrounding bone when the bone-implant complex is subjected to loading, shown for the Control (blank) group, pure Ti group, and experimental Mg-Ti composites groups (Mg-Ti-900, Mg-Ti-1200, Mg-Ti-1500). (B) FEA of stress distribution on the implant materials, illustrating the long axis (shaft) and head regions for the Ti group and Mg-Ti composites (Mg-Ti-900, Mg-Ti-1200, Mg-Ti-1500).
